# Association of Hypertension and Diabetes with Ischemic Heart Disease and Stroke Mortality in India: The Million Death Study

**DOI:** 10.5334/gh.1048

**Published:** 2021-10-14

**Authors:** Calvin Ke, Rajeev Gupta, Baiju R. Shah, Thérèse A. Stukel, Denis Xavier, Prabhat Jha

**Affiliations:** 1 Centre for Global Health Research, Unity Health, and Dalla Lana School of Public Health, University of Toronto, Toronto, CA; 2 Department of Medicine, University of Toronto, Toronto, CA; 3 Institute of Health Policy, Management and Evaluation, University of Toronto, Toronto, CA; 4 ICES, University of Toronto, Toronto, CA; 5 Development Unit, Rajasthan University of Health Sciences, Jaipur, Rajasthan, IN; 6 St. John’s Medical College and Research Institute, Bangalore, IN

**Keywords:** cardiovascular disease, mortality, epidemiology, nationally representative

## Abstract

**Background:**

The cardiovascular outcomes of hypertension and diabetes in India have never been studied at the national level.

**Objectives:**

We conducted a nationally-representative proportional mortality study to measure the associations of hypertension and diabetes with premature mortality due to ischemic heart disease (IHD) and stroke among Indian adults.

**Methods:**

We determined causes of death by verbal autopsy from 2001–14 among 2.4 million households. We defined cases as those who died of the study outcomes and controls as those who died of injuries, respiratory causes, or cancer. We used multivariable logistic regression models to compute adjusted odds ratios (OR) measuring the association of hypertension and diabetes with IHD or stroke mortality, population-attributable fractions (PAF), and time trends.

**Results:**

The mean age at death was 55.6 (standard deviation 9.9) years for IHD, 58.2 (9.0) years for stroke, and 46.8 (injury) to 59.8 (respiratory) years for controls. There were more men among both the cases (IHD: 70.1%; stroke: 59.0%) and controls (injury: 76.6%; cancer: 55.4%; respiratory: 59.8%). Hypertension was associated with six- to eight-fold increases in the odds of IHD (OR 5.9, 99% CI 5.6–6.2) and stroke mortality (7.9, 7.4–8.5). Diabetes was associated with double the odds (1.9, 1.7–2.0) of IHD mortality and increased odds of stroke mortality (1.6, 1.4–1.7). Hypertension accounted for an increasing PAF of IHD mortality and decreasing PAF of stroke mortality. Diabetes was associated with relatively lower PAFs and variable time trends.

**Conclusions:**

Hypertension is associated with an unexpectedly high burden of cardiovascular mortality, and contributes to an increasing proportion of IHD deaths and a decreasing proportion of stroke deaths. Better management of hypertension and diabetes is urgently required to reduce premature cardiovascular mortality.

## Introduction

Cardiovascular disease causes an estimated 17.7 million deaths every year [1]. These deaths consist primarily of ischemic heart disease and stroke [2]. India accounts for over one-fifth of premature cardiovascular deaths worldwide [2]. We previously reported that cardiovascular mortality rates diverged substantially in India over the past two decades [2]. Ischemic heart disease mortality rates in India have risen, particularly in rural areas—increasing by approximately 50% from 2000 to 2015 to exceed the urban rates [2]. By contrast, stroke mortality rates dropped by up to a third over this period, falling faster in urban than in rural areas [2].

The causes of these divergent patterns have never been identified due to the scarcity of longitudinal, nationally-representative data on cardiovascular risk factors and outcomes in India. In the context of unprecedented urbanization and economic growth, the prevalence rates of hypertension and diabetes have dramatically increased [3]. One in four Indian adults has hypertension, and 7.5% have diabetes [3]. Yet, the associations of hypertension and diabetes with cardiovascular mortality have only been studied in small, local populations [4,5,6]. The contributions of these risk factors to the changing trends in cardiovascular mortality have never been characterized. Furthermore, the burdens of hypertension and diabetes in India likely differ from high-income countries due to relatively poor control [3], and disparities in health services and case fatality rates [7]. We conducted a nationally-representative proportional mortality study to measure the associations and population-attributable fractions of hypertension and diabetes with premature mortality due to ischemic heart disease and stroke among Indian adults in the 14-year Million Death Study.

## Methods

### Study Setting and Data Source

In India, most deaths occur at home without medical certification. The Registrar General of India established the Sample Registration System in 1971 to monitor causes of death [8]. The Registrar General partitions India into 1 million units consisting of 150–300 households each, and selects a random sample of units to monitor for deaths. This sampling frame is re-created after every decennial census. The 6,671 and 7,597 units selected after the 1991 and 2001 censuses comprise the 2.4 million households participating in the Million Death Study (1991 census: 2001–03; 2001 census: 2004–14) [9]. This sample is demographically representative of the national population of India.

International cardiovascular epidemiological guidelines recommend using verbal autopsy to diagnose the cause of death in low-income settings where standard death certification is unavailable [10]. A multicentre validation study in India showed that verbal autopsy has a 75.0% sensitivity and 92.7% specificity for identifying ischemic heart disease deaths, and a 75.0% sensitivity and 95.0% specificity for identifying stroke deaths among adults [11]. To improve classification, we restricted the deaths to age 30–69 years, as the proportion of ill-defined deaths is greater after age 70 years (ill-defined deaths: 18% [age >70 years], <4% [age <70 years]) [12]. Each death was investigated by one of 900 specially-trained non-medical surveyors deployed every six months. The verbal autopsy consisted of an interview with a household member of the deceased using a modified version of the World Health Organization questionnaire to collect demographic and clinical information including pre-existing conditions diagnosed by a physician [2]. Surveyors were also trained to elicit a detailed narrative of events preceding each death using a standard list of cardinal symptoms. Each narrative was recorded in the local language and reviewed independently by two of 400 physicians, matched according to language ability. Each physician was specially trained to determine the most probable cause of death according to strict guidelines based upon the symptoms preceding death. Myocardial infarction was defined as an episode of severe chest pain lasting 30 minutes to 24 hours with shortness of breath, vomiting, or left arm pain [2,11]. Stroke was defined as sudden onset of paralysis of one or more limbs with altered speech, loss of sensation, or loss of vision, within one month preceding death [2,11]. Physicians encoded each diagnosis using the International Statistical Classification of Diseases and Related Health Problems version 10 (eTable 1). In case of disagreement, each physician anonymously reviewed the other’s diagnostic code and rationale. Persisting disagreements were adjudicated by a third senior physician. There were extensive procedures to ensure a high quality of fieldwork and physician coding [12]. A random selection of 4043 (5%) deaths occurring in 2002–03 were independently re-surveyed and re-reviewed, and the results were highly consistent (test-retest odds ratio 1.0 [95% confidence interval 0.9–1.2] for ischemic heart disease and stroke) [12].

### Study Design and Population

We used the proportional mortality study design, which is a variant of the case-control study [13,14]. Cases are those who died of an outcome of interest, and controls are those who died of a cause unrelated to the exposure of interest. The primary exposures were physician-diagnosed hypertension and diabetes. Each of these exposures was specifically elicited from respondents by the trained surveyors using a standardized checklist [12]. Although these exposures may have been recalled inaccurately by some respondents, we minimized the effect of misclassification bias by ascertaining the exposure status in an identical manner among both cases and controls [15]. This methodology has been applied in many similar studies [14,16,17]. The outcomes were death due to ischemic heart disease or stroke among adults aged 30–69 years during 2001–14 [18]. Based on our previous findings of unusually elevated stroke mortality among a cluster of states in northeastern India (Appendix), we analyzed stroke in these ‘high-burden’ states separately from the remaining ‘low-burden’ states [2]. We defined controls as those who died of injuries (eTable 1). As injury deaths comprised mostly of young men, we added respiratory and cancer deaths as alternative control groups to provide more comparable age and sex distributions.

### Statistical Analysis

We described baseline characteristics among the cases and controls. We used multivariable logistic regression models to measure the association of hypertension or diabetes with ischemic heart disease or stroke mortality. We adjusted for pre-specified covariates identified based on clinical significance, including age, sex, urban or rural residence, region, smoking, and alcohol use. We computed adjusted odds ratios (ORs) with 99% confidence intervals (CI; as per Million Death Study conventions) separately for each control group and for all the controls pooled together. We used these ORs to generate population attributable fractions (PAFs) [19]. Given the long study duration, we tested whether the effects of hypertension and diabetes varied by year. These interaction terms were all significant, except for diabetes in high-burden stroke states (Appendix). We included the significant interaction terms and centred at the midpoint of the study period. To facilitate local health planning, we also reported the main findings by state.

We conducted extensive sensitivity analyses to test the validity of our assumptions. To examine whether cause of death misclassification may have affected the results, we repeated all analyses restricting to deaths where both physician adjudicators immediately agreed on the cause of death category. To examine whether diabetes and hypertension may have interacted unexpectedly, we repeated the primary analysis categorizing the exposures as diabetes alone, hypertension alone, and both diabetes and hypertension. To determine whether socioeconomic status may have meaningfully affected our findings, we repeated all analyses adjusting for socioeconomic status quintile using district-level light emissions at night as a proxy variable (restricted to 2001–13 based on data availability) [20]. Finally, we repeated all analyses adjusting for clustering at the district level. We also explored the association between the use of any regular medications within five years prior to death (respondent-reported) and the study outcomes among people with hypertension only, diabetes only, and both. Variables with unknown values were assigned to a separate category. Missing covariate data were minimal (Table 1) and handled by complete case analysis. We used SAS version 9.4 for all analyses. The Million Death Study was approved by the Indian Council of Medical Research. The study protocol was approved by the research ethics boards of St. Michael’s Hospital and University of Toronto.

**Table 1:** Baseline characteristics of case and control populations aged 30 to 69 years in the Million Death Study (2001–14). All values in the table are percentages unless otherwise indicated.

Characteristics	Cases				Controls		

	Ischemic Heart Disease	Stroke			Injury	Cancer	Respiratory

		High-Burden	Low-Burden	Total			

	n = 45,230	n = 8,176	n = 14,275	n = 22,451	n = 15,899	n = 22,197	n = 22,353

Mean age (years; standard deviation)	55.6 (9.9)	57.4 (9.2)	58.7 (8.8)	58.2 (9.0)	46.8 (11.4)	54.2 (10.1)	59.8 (8.3)
Age Group (years)							
30–34	1,525 (3.4)	149 (1.8)	245 (1.7)	394 (1.8)	2,684 (16.9)	916 (4.1)	309 (1.4)
35–39	2,205 (4.9)	305 (3.7)	385 (2.7)	690 (3.1)	2,615 (16.4)	1,374 (6.2)	432 (1.9)
40–44	3,344 (7.4)	452 (5.5)	618 (4.3)	1,070 (4.8)	2,199 (13.8)	2,038 (9.2)	768 (3.4)
45–49	4,639 (10.3)	695 (8.5)	915 (6.4)	1,610 (7.2)	2,056 (12.9)	2,585 (11.6)	1,146 (5.1)
50–54	6,260 (13.8)	1,060 (13.0)	1,572 (11.0)	2,632 (11.7)	1,809 (11.4)	3,294 (14.8)	2,177 (9.7)
55–59	7,966 (17.6)	1,448 (17.7)	2,346 (16.4)	3,794 (16.9)	1,644 (10.3)	3,854 (17.4)	3,584 (16.0)
60–64	9,321 (20.6)	1,889 (23.1)	3,565 (25.0)	5,454 (24.3)	1,472 (9.3)	4,110 (18.5)	5,819 (26.0)
65–69	9,970 (22.0)	2,178 (26.6)	4,629 (32.4)	6,807 (30.3)	1,420 (8.9)	4,026 (18.1)	8,118 (36.3)
Women	13,518 (29.9)	3,694 (45.2)	5,513 (38.6)	9,207 (41.0)	3,721 (23.4)	9,896 (44.6)	8,978 (40.2)
Residence							
Rural	31,752 (70.2)	5,589 (68.4)	11,101 (77.8)	16,690 (74.3)	12,406 (78.0)	16,696 (75.2)	18,387 (82.3)
Urban	13,478 (29.8)	2,587 (31.6)	3,174 (22.2)	5,761 (25.7)	3,493 (22.0)	5,501 (24.8)	3,965 (17.7)
Socioeconomic Status Quintile (Q, 2001–13 only)							
Q1 (lowest)	5,276 (12.6)	1,504 (19.7)	2,466 (18.2)	3,970 (18.7)	2,810 (18.9)	3,591 (17.1)	5,453 (25.6)
Q2	6,729 (16.0)	1,877 (24.6)	2,698 (19.9)	4,575 (21.6)	2,872 (19.3)	4,073 (19.5)	4,781 (22.5)
Q3	8,632 (20.6)	1,403 (18.4)	2,938 (21.7)	4,341 (20.5)	3,005 (20.2)	4,280 (20.4)	4,357 (20.5)
Q4	10,112 (24.1)	1,154 (15.1)	2,848 (21.0)	4,002 (18.9)	3,205 (21.6)	4,603 (22.0)	3,581 (16.8)
Q5 (highest)	11,201 (26.7)	1,691 (22.2)	2,608 (19.2)	4,299 (20.3)	2,969 (20.0)	4,393 (21.0)	3,108 (14.6)
Education (2001–3 only)							
Illiterate	3,096 (42.9)	667 (50.3)	1,726 (57.2)	2,393 (55.1)	1,127 (42.3)	1,905 (48.5)	3,608 (71.2)
Primary or below	2,025 (28.1)	372 (28.1)	780 (25.8)	1,152 (26.5)	700 (26.3)	1,102 (28.0)	964 (19.0)
Above primary	1,360 (18.9)	202 (15.2)	357 (11.8)	559 (12.9)	576 (21.6)	653 (16.6)	365 (7.2)
Missing	733 (10.2)	84 (6.3)	156 (5.2)	240 (5.5)	263 (9.9)	270 (6.9)	131 (2.6)
Occupation (2001–3 only)							
Unemployed	3,133 (43.4)	689 (52.0)	1,538 (50.9)	2,227 (51.3)	878 (32.9)	1,834 (46.7)	2,499 (49.3)
Agricultural	1,618 (22.4)	225 (17.0)	674 (22.3)	899 (20.7)	716 (26.9)	944 (24.0)	1,452 (28.7)
Wage	655 (9.1)	97 (7.3)	275 (9.1)	372 (8.6)	377 (14.1)	362 (9.2)	500 (9.9)
Salaried	690 (9.6)	107 (8.1)	183 (6.1)	290 (6.7)	262 (9.8)	273 (6.9)	139 (2.7)
Professional/Business	620 (8.6)	83 (6.3)	178 (5.9)	261 (6.0)	258 (9.7)	260 (6.6)	214 (4.2)
Other	494 (6.8)	124 (9.4)	171 (5.7)	295 (6.8)	174 (6.5)	252 (6.4)	263 (5.2)
Death Place							
Home	29,234 (64.6)	5,479 (67.0)	10,698 (74.9)	16,177 (72.1)	5,300 (33.3)	16,153 (72.8)	18,395 (82.3)
Health facility	11,455 (25.3)	2,240 (27.4)	2,883 (20.2)	5,123 (22.8)	4,096 (25.8)	5,092 (22.9)	2,942 (13.2)
Other	3,511 (7.8)	273 (3.3)	361 (2.5)	634 (2.8)	6,089 (38.3)	427 (1.9)	480 (2.1)
Unknown	1,030 (2.3)	184 (2.3)	333 (2.3)	517 (2.3)	414 (2.6)	525 (2.4)	536 (2.4)
Major Region							
North	7,656 (16.9)		1,861 (13.0)	1,861 (8.3)	2,355 (14.8)	3,257 (14.7)	3,029 (13.6)
Central	5,861 (13.0)	536 (6.6)	2,349 (16.5)	2,885 (12.9)	3,054 (19.2)	3,403 (15.3)	5,988 (26.8)
Northeast	1,690 (3.7)	2,653 (32.4)		2,653 (11.8)	1,068 (6.7)	2,262 (10.2)	1,278 (5.7)
East	6,533 (14.4)	4,987 (61.0)	1,936 (13.6)	6,923 (30.8)	2,912 (18.3)	3,702 (16.7)	4,097 (18.3)
West	6,750 (14.9)		2,496 (17.5)	2,496 (11.1)	1,914 (12.0)	2,800 (12.6)	2,768 (12.4)
South	16,740 (37.0)		5,633 (39.5)	5,633 (25.1)	4,596 (28.9)	6,773 (30.5)	5,193 (23.2)
Previous Comorbidities*							
Diabetes	6,056 (13.7)	967 (11.9)	1,826 (12.3)	2,793 (12.1)	273 (2.7)	958 (4.7)	875 (3.8)
Hypertension	16,113 (36.0)	3,665 (44.6)	5,787 (39.9)	9,452 (41.7)	549 (5.2)	1,914 (9.1)	2,128 (9.2)
Heart Disease	25,513 (56.3)	1,445 (17.7)	1,327 (9.4)	2,772 (12.4)	300 (2.3)	659 (3.2)	1,048 (4.7)
Stroke	4,478 (10.0)	4,854 (60.9)	7,736 (53.1)	12,590 (55.9)	184 (1.6)	336 (1.6)	290 (1.3)
Cancer	240 (0.5)	48 (0.6)	92 (0.7)	140 (0.6)	57 (0.4)	18,378 (83.0)	217 (1.0)
Asthma	2,619 (6.0)	540 (6.6)	743 (5.0)	1,283 (5.6)	289 (2.8)	1,176 (5.7)	18,372 (81.4)
Medications (2004–14 only)*							
Taking any medication	8,819 (23.6)	1,339 (19.6)	3,881 (33.7)	5,220 (28.3)	672 (6.7)	7,193 (39.6)	5,923 (34.4)
Missing	3,773 (9.9)	608 (8.8)	1,268 (11.2)	1,876 (10.3)	1,440 (10.8)	1,927 (10.6)	1,766 (10.4)
Smoking (men only)*							
None	17,569 (55.6)	2,155 (47.7)	5,103 (58.1)	7,258 (54.4)	7,047 (57.9)	5,551 (45.1)	5,904 (43.8)
Cigarettes only	2,436 (7.6)	334 (7.7)	379 (4.5)	713 (5.6)	752 (5.1)	996 (8.0)	474 (3.8)
Bidis only	8,231 (25.9)	1,341 (29.8)	2,535 (28.6)	3,876 (29.0)	2,961 (26.1)	3,948 (32.3)	5,333 (39.5)
Cigarettes and bidis	2,477 (7.7)	538 (12.2)	449 (5.4)	987 (7.8)	932 (7.1)	1,446 (11.7)	1,199 (9.4)
Missing	999 (3.2)	114 (2.6)	296 (3.3)	410 (3.1)	486 (3.7)	360 (2.9)	465 (3.5)
Alcohol Use (days per week, men only)*							
None	21,565 (68.4)	3,281 (72.4)	5,958 (67.1)	9,239 (68.8)	7,788 (66.0)	7,950 (65.1)	9,382 (68.7)
1–4	5,100 (15.9)	523 (11.9)	1,471 (17.1)	1,994 (15.4)	2,090 (16.5)	1,932 (15.6)	2,036 (15.8)
5–7	3,117 (9.6)	412 (9.6)	855 (10.2)	1,267 (10.0)	1,436 (11.0)	1,596 (12.8)	1,097 (8.9)
Missing	1,346 (4.2)	169 (3.9)	346 (4.0)	515 (4.0)	592 (4.6)	517 (4.2)	623 (4.7)

Abbreviations: Q, quintile. * Percentages adjusted to the age and sex distribution of all cases. See eTable 1 for unadjusted percentages and unknown values.

## Results

The baseline characteristics are shown in Table 1 (eTable 2). The mean age was 55.6 (standard deviation 9.9) years for ischemic heart disease and 58.2 (9.0) years for stroke deaths. The mean age of control deaths ranged from 46.8 (injury) to 59.8 (respiratory) years. There were more men among both the cases (ischemic heart disease: 70.1%; stroke: 59.0%) and controls (injury: 76.6%; cancer: 55.4%; respiratory: 59.8%). Most deaths occurred in rural areas (68.4–82.3%) at home (64.6–82.3%). Ischemic heart disease had the highest socioeconomic status distribution (26.7% highest quintile), and respiratory deaths had the lowest (25.6% lowest quintile). Illiteracy was most common among respiratory deaths (71.2%), while unemployment was most common among stroke deaths (51.3%). Diabetes prevalence varied from 11.9% (high-burden stroke) to 13.7% (ischemic heart disease) among cases, and from 2.7% (injuries) to 4.7% (cancer) among controls (percentages were adjusted to match the age and sex distribution of the cases; see eTable 2 for unadjusted percentages). Reported hypertension prevalence varied from 36.0% (high-burden stroke) to 44.6% (ischemic heart disease) among cases, and from 5.2% (injuries) to 9.2% (respiratory) among controls. Over half of the cases reported previous heart disease (56.3%) or a previous stroke (55.9%). Medication use was uncommon among cases (23.6% for ischemic heart disease, 28.3% for stroke) and varied among controls (range: 6.7% for injury, 39.6% for cancer). Bidi smoking (a local form of tobacco use) was most common among male high-burden stroke (29.8%), cancer (32.3%), and respiratory deaths (39.5%). Alcohol use was uncommon.

**Figure 1:**
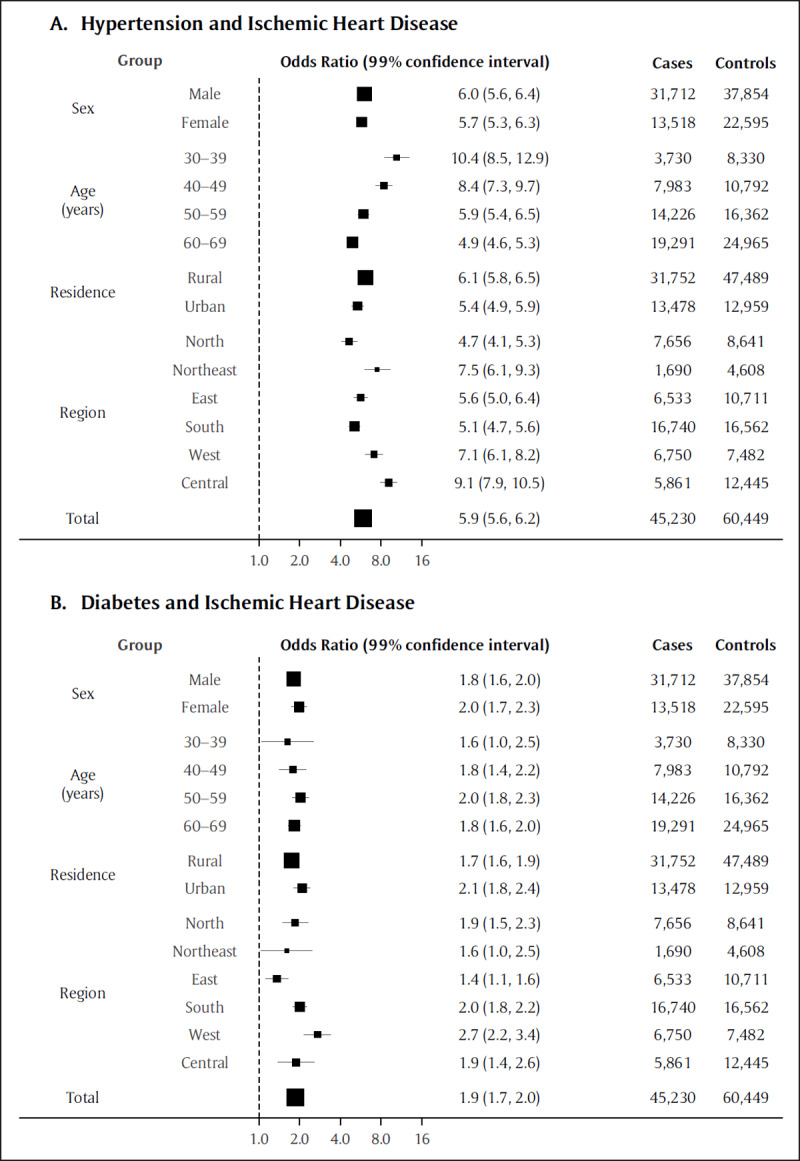
Total and stratified estimates of the association between **(A)** hypertension or **(B)** diabetes and ischemic heart disease mortality. Estimates are adjusted for age, sex, rurality, region, smoking, alcohol use, and year. The area of each box is proportional to the sample size (cases and controls).

Figure 1 shows the association of hypertension and diabetes with ischemic heart disease mortality. These ORs pertain to all controls pooled together; the results for separate control groups were all consistent in direction (eFigures 1–3). Hypertension was associated with a six-fold increase (OR 5.9, 99% CI 5.6–6.2, p < 0.0001) in the odds of ischemic heart disease mortality. Stratified estimates revealed an inverse age gradient, with the strongest association among young adults (10.4, 8.5–12.9 for ages 30–39 years, p < 0.0001). Geographically, the highest ORs were in the northeast (7.5, 6.1–9.3, p < 0.0001) and central (9.1, 7.9–10.5, p < 0.0001) regions. Diabetes was associated with double the odds (1.9, 1.7–2.0, p < 0.0001) of ischemic heart disease mortality. There were geographic differences, with the highest OR in western states (2.7, 2.2–3.4, p < 0.0001). The remaining stratified estimates were consistent with the overall estimate.

**Figure 2:**
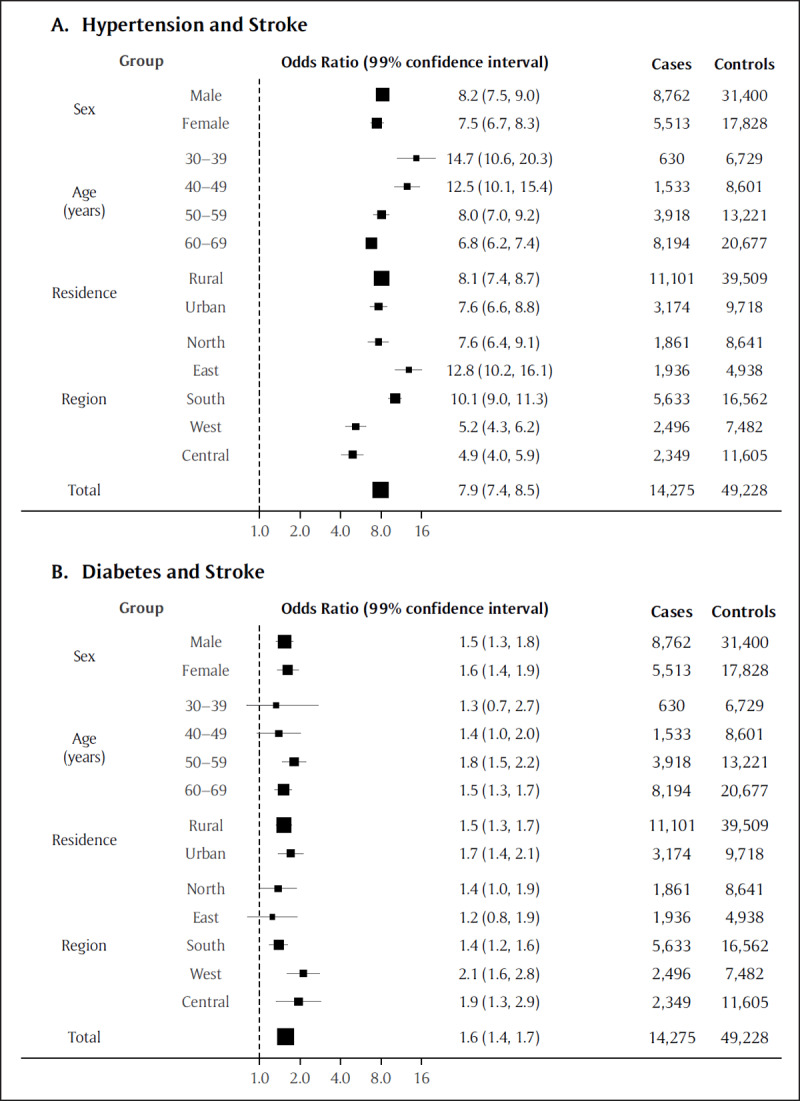
Association between hypertension **(A)** or diabetes **(B)** and stroke* mortality. The total and stratified estimates are adjusted for age, sex, urban/rural residence, region, smoking, alcohol use, and year. The area of each box is proportional to the sample size (cases and controls). * These results pertain to the low-burden states; see Figure 4 for the high-burden states.

Hypertension was similarly associated with an eight-fold increase in the odds of stroke mortality in low-burden states (Figure 2; 7.9, 7.4–8.5, p < 0.0001), with an inverse age gradient peaking among adults aged 30–39 years (14.7, 10.6–20.3, p < 0.0001). Associations were stronger in the eastern 12.8 (10.2–16.1, p < 0.0001) and southern (10.1, 9.0–11.3, p < 0.0001) states. Diabetes was associated with a significantly increased odds of stroke mortality (1.6, 1.4–1.7, p < 0.0001), with generally consistent estimates across strata. Findings in high- and low-burden states appeared mostly similar, but stratification by region revealed far higher ORs for hypertension in the northeast (14.0, 11.6–17.0, p < 0.0001) compared with the east (4.7, 4.1–5.4, p < 0.0001) and central (5.5, 2.5–12.1, p < 0.0001) high-burden states (eFigure 4).

**Figure 3:**
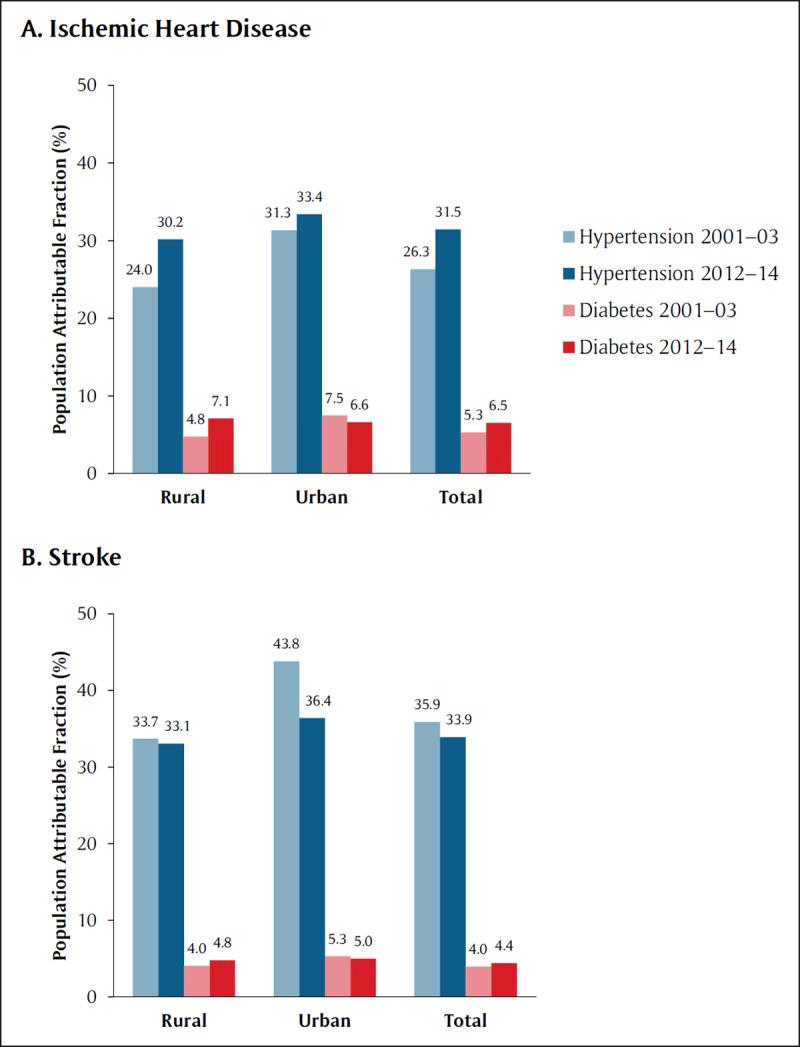
Population attributable fractions (%) for **(A)** ischemic heart disease and **(B)** stroke* during the beginning (2001–03) and end (2012–14) of the study period. Blue bars represent hypertension; red bars represent diabetes. * These results pertain to the low-burden states; see Figure 5 for the high-burden states.

Figure 3 shows the estimated PAFs from the beginning to the end of the study. Hypertension was associated with increasing PAFs over time for ischemic heart disease mortality (total: 31.5%, 2012–14), with a greater increase in rural versus urban areas. By contrast, hypertension was associated with decreasing PAFs over time for stroke mortality in the low-burden states (total: 33.9%, 2012–14), with a greater decrease in urban versus rural areas. Diabetes was associated with relatively lower PAFs (1.7–7.5%) with variable trends over time. PAFs in high-burden states were generally similar to low-burden states (eFigure 5). Additional analyses categorizing the exposures as diabetes alone and hypertension alone resulted in similar ORs to the main analysis, and the combination of diabetes and hypertension was associated with similar ORs compared to hypertension alone (eFigure 4). ORs varied in magnitude across states (eTable 3). The remaining sensitivity analyses were consistent with the main analysis (eFigures 6–17). In an exploratory analysis, respondent-reported medication use was associated with decreased ischemic heart disease and stroke (high-burden) mortality among people with hypertension (eFigure 18, eTable 4).

## Discussion

In this nationally-representative study, we revealed that hypertension is associated with an unexpectedly high burden of cardiovascular disease in India, with six- to eight-fold elevations in the odds of ischemic heart disease and stroke mortality across urban and rural areas. Furthermore, we revealed that hypertension contributes to an increasing proportion of ischemic heart disease deaths and a decreasing proportion of stroke deaths—a novel finding suggesting that the changing burden of hypertension has likely shaped the divergent patterns in cardiovascular mortality over the past two decades. Moreover, there was considerable heterogeneity across regions, with relatively strong associations between hypertension and stroke in the northeastern states, which may require targeted interventions. Compared to high-income countries, these results emphasize the especially important role of hypertension in shaping cardiovascular outcomes across India. Diabetes was associated with up to double the odds of mortality due to ischemic heart disease and stroke, which is consistent with evidence from other countries. These real-world findings suggest that better management of hypertension and diabetes is urgently required in low- and middle-income countries to reduce premature cardiovascular mortality, especially from ischemic heart disease.

Hypertension was associated with six to eight times the odds of cardiovascular mortality—a particularly strong relationship compared with the international Prospective Urban Rural Epidemiology study (PURE; hazard ratio 1.9 [1.6–2.2] for cardiovascular mortality in low-income countries, mostly [78.8%] from India) and other small, local studies [4,5,6]. The reason for these differences is unknown, but disparities in blood pressure control, cardiovascular event incidence, and case fatality rates likely play a role. Every 20 mm Hg increase in systolic blood pressure doubles the risk of ischemic heart disease mortality and more than doubles the risk of stroke mortality [21]. However, only 7.9% (7.6–8.3%) of Indians with hypertension have adequate blood pressure control—a rate that is much lower than high-income countries and similar to other low- and middle-income countries [22,23,24]. Better rates of hypertension control among the Indian and low-income country participants of the PURE study (12.7–23.4%) suggest that selection bias in PURE likely resulted in lower hazard ratios [25]. Furthermore, the INTERSTROKE case-control study of 22 low- to high-income countries reported that hypertension was a stronger risk factor for stroke incidence among South Asians (OR 3.9, 99% CI 3.1–4.9) than Europeans (2.0, 1.6–2.5)—a finding supported by multiethnic studies of South Asians in high-income countries [4,26]. Finally, gaps in treatment and health services likely contribute to higher case fatality rates in India than in high-income countries [7].

Furthermore, the PAFs of hypertension diverged strikingly over time for ischemic heart disease and stroke deaths, and these differences likely explain the divergent trends in cardiovascular mortality that we previously reported [2]. The magnitude of the PAFs of hypertension and diabetes that we observed appeared higher and lower respectively than those reported for overall cardiovascular deaths across all countries in the PURE study (results for India not specifically reported) [5], although these comparisons should be interpreted with caution due to methodological differences across studies [27]. For hypertension and ischemic heart disease, the PAF increased in rural areas more than in urban areas, which corresponds with the rapidly rising ischemic heart disease mortality rates in rural areas [2]. Conversely, the PAFs for stroke dropped more in urban than in rural areas, which is consistent with stroke mortality rates dropping faster in urban than rural areas [2]. However, it is unclear why these PAFs changed in different directions for ischemic heart disease and stroke. While the prevalence of hypertension appears to be generally rising in India [28], it is well-established that severe hypertension is a stronger risk factor for stroke than for ischemic heart disease [29], and the frequency of severe hypertension may decrease with economic development [30].

Our study is also the first to demonstrate important differences in the association between hypertension and cardiovascular outcomes across states, with especially strong associations between hypertension and stroke in Andhra Pradesh (a southeastern state with a human development index similar to Nicaragua; 29.2, 22.4–38.0), Assam (a northeastern state with a human development index similar to Bangladesh; 23.6, 17.3–32.2), and the other northeast states (9.3, 7.2–11.9). In Assam, high salt intake may also cause more severe or uncontrolled hypertension [31], which likely increases stroke risk [29]. Despite the strong associations of hypertension and stroke in the high-burden states, the PAFs for these states appeared similar to the rest of the country. Further study is required to understand how other risk factors might explain the disproportionately high stroke mortality rates reported in these states [2].

By contrast, diabetes was associated with double the odds of ischemic heart disease death and 1.2 to 1.6 times the odds of stroke death. These results are lower than the rate ratios reported in high-income countries (overall cardiovascular mortality: 2.2, 2.0–2.3) [32], low-income countries (2.1, 1.8–2.6) [5], as well as in China and Mexico for ischemic heart disease (China: 2.4, 2.2–2.6 [33]; Mexico: 3.7, 3.2–4.2 [34]) and stroke mortality in particular (China: 2.0, 1.8–2.2; Mexico: 3.5, 3.0–4.2). In some, but not all [35], multiethnic studies from high-income nations [36], Indian or South Asian ethnicity is associated with a similar or lower hazard of cardiovascular complications or mortality after diabetes diagnosis relative to Europeans [37]. Other studies from high-income nations have shown that diabetes is associated with an approximately 50% higher risk of cardiovascular mortality among women than men [32,38,39]. Interestingly, we observed that diabetes was associated with similar odds of cardiovascular mortality among men and women—a finding also described in Mexico [34]. In India, only 20.8% of men and 29.6% of women with diabetes achieve glycemic control [40]. Relatively better glycemic control among Indian women might stem from gestational diabetes management practies [40]. However, glycemic control among Mexican women appears to be similar to or worse than Mexican men [34,41]. Further research is needed to investigate how sex influences cardiovascular disease among Indians [42,43].

### Strengths and Limitations

This study provides the first nationally representative evidence directly characterizing the relationship between hypertension and diabetes with cardiovascular mortality in India. Our large sample allowed us to characterize important subnational variations using a statistically efficient proportional mortality methodology, and the long study duration yielded informative insights into the changing burden of hypertension and diabetes over time. The rigorous study protocol, extensive quality control measures, and consistent sensitivity analyses support the validity of our findings. However, there are some limitations to note. Undiagnosed hypertension was likely more common among the apparently healthy injury controls, thus resulting in higher ORs. Nevertheless, we included respiratory and cancer deaths as additional controls to ensure a balanced age and sex distribution, and the majority of pooled controls had previously been assessed by a physician for various conditions, thus reducing the possibility of exposure misclassification bias. We lacked blood pressure and laboratory data, and we cannot rule out the presence of residual confounding by unmeasured variables or recall bias. We had no autopsy or neuroimaging data because most deaths occurred at home. However, verbal autopsy is a reliable and accurate method that is widely recommended for use in low-income countries by international cardiovascular guidelines [10,11,12], and our sensitivity analysis restricted to deaths where both physicians immediately agreed on the cause of death showed similar results.

## Conclusions

In summary, our study illustrates how hypertension and diabetes contribute to the divergent trends in ischemic heart disease and stroke mortality in India [2]. Achieving the United Nations Sustainable Development Goal to reduce cardiovascular mortality by one third will require substantial expansion of services for hypertension and diabetes in India by continued investment in the upgrading and construction of 150,000 Health and Wellness Centres to improve primary care services, and by addressing treatment delays arising from lack of awareness [22], inequitable access to health care services, and high drug costs. Considering the low rates of preventive medication use that we and others have reported previously [2,22], future policies to strengthen health systems and empower primary care providers and non-physician health workers will be required to achieve universal health care and reduce the disproportionate burden of cardiovascular disease in India [28].

## Data Accessibility Statement

Data from the Million Death Study cannot be redistributed outside of the Centre for Global Health Research due to a legal agreement with the Registrar General of India. However, access to Million Death Study data can be granted via data transfer agreements, upon request to the Office of the Registrar General, R. K. Puram, New Delhi, India (rgi.rgi@nic.in).

## Additional File

The additional file for this article can be found as follows:

10.5334/gh.1048.s1 Appendix. Additional Methodology and Results.
