# Supplementary material for: Association of Hypertension and Diabetes with Ischemic Heart Disease and Stroke Mortality in India: The Million Death Study
Source: Glob Heart. 2021 Oct 14;16(1):69. doi: 10.5334/gh.1048 (PMC8516008; doi:10.5334/gh.1048)
Supplement: Appendix. — Additional Methodology and Results. [file gh-16-1-1048-s1.pdf]

Appendix

I. Additional Methodology

eTable 1 Causes of death in the Million Death Study, categorized using 3-character codes from the *International Statistical Classification of Diseases and Related Health Problems* version 10 (ICD-10). The estimated sensitivity and specificity of verbal autopsy is indicated for each cause of death.[1]

Cause of Death	ICD-10 Codes	Sensitivity (%)	Specificity (%)
Ischemic heart disease	I20–I25, I46, R55, R96	75.0	92.7
Stroke	G45–G46, I60–I67, I69, G81–83	75.0	95.0
Injury	D53, F79, K46, N43, Q20, Q21, Q24, V02, V03, V04, V05, V09, V12, V13, V14, V18, V23, V24, V26, V27, V28, V29, V33, V34, V38, V39, V44, V47, V48, V49, V54, V58, V59, V64, V68, V69, V78, V79, V80, V81, V84, V87, V89, V98, V99, W11, W14, W15, W20, W23, W36, W54, W55, W57, W69, W70, W74, W77, W79, W85, W86, W87, X00, X02, X04, X06, X08, X09, X20, X30, X32, X33, X44, X45, X48, X49, X91, X94, X95, X99, Y00, Y04, Y08, Y09, Y86, Y88, Z88	84.9	96.0
Cancer	C16, C14, C26, C22, C95, C55, C34, C10, C71, C80, C15, C06, C76, C32, C40, C41, C18, C39, C64	61.1	96.0
Respiratory	J45, J18, J44	21.4	97.0

Cases

In accordance with local coding conventions, I46 (cardiac arrest), R55 (syncope and collapse), and R96 (other sudden death, cause unknown) were included in the definition of ischemic heart disease. In practice, these adjustments made little absolute difference because the vast majority of premature mortality in this category were classified under I21 (88·9%), I22 (3·9%), and I25 (3·0%).

Similarly, we included codes for hemiplegia and other paralytic syndromes (G81–G83) based on local coding conventions. In practice, this adjustment made little absolute difference because the vast majority of deaths in this category were classified under I64 (72·9%), I69 (17·1%), and I61 (6·5%).

Controls

After excluding deaths due to ischemic heart disease and stroke, we examined the remaining causes of death with at least 50 cases each. We eliminated any causes of death with a known etiological association with hypertension or diabetes based on a review of the literature. We first grouped together the causes of death related to injury. As injury deaths consisted predominantly of young men, we defined two additional control groups dying of cancer and respiratory conditions, which were the most common remaining causes of death. We pooled the control groups together for the main estimates, and generated additional estimates using each control group separately.

High-Burden States

There is a cluster of states in northeastern and eastern India that where the rate of stroke mortality is three times greater higher than the national average.[2] We characterized these states as "high-burden" states. Because cardiovascular risk factor distributions might be different in these states, we analyzed this cluster separately for the stroke outcomes. The high-burden states are: Assam, West Bengal, Chhattisgarh, Sikkim, Arunachal Pradesh, Nagaland, Manipur, Mizoram, Tripura, and Meghalaya, for men and women; Odisha for women only.

Time Trends

We tested whether the effects of hypertension and diabetes varied by year. For ischemic heart disease, there was a significant interaction between hypertension and year (p=0.0002) and diabetes and year (p=0.0001). For stroke, there was a significant interaction between hypertension and year in both high- and low-burden states (p=0.002 and p=0.01 respectively), and between diabetes and year in low-burden states only (p=0.002). These interaction terms were included in all logistic regression models.

II. Additional Results

eTable 2 Baseline characteristics of case and control populations aged 30 to 69 years in the Million Death Study (2001 to 2014). All values in the table are unadjusted percentages unless otherwise indicated. Missing values were <0.5% unless otherwise indicated.

Characteristics	Cases				Controls		
	Ischemic Heart Disease (n=45,230)	Stroke			Injury (n=15,899)	Cancer (n=22,197)	Respiratory (n=22,353)
		High-Burden (n=8,176)	Low-Burden (n=14,275)	Total (n=22,451)			
Previous Comorbidities							
Diabetes	6,056 (13.4)	967 (11.8)	1,826 (12.8)	2,793 (12.4)	273 (1.7)	958 (4.3)	875 (3.9)
Unknown	3,299 (7.3)	947 (11.6)	1,004 (7.0)	1,951 (8.7)	1,290 (8.1)	1,798 (8.1)	1,834 (8.2)
Hypertension	16,113 (35.6)	3,665 (44.8)	5,787 (40.5)	9,452 (42.1)	549 (3.5)	1,914 (8.6)	2,128 (9.5)
Unknown	3,006 (6.6)	697 (8.5)	891 (6.2)	1,588 (7.1)	1,316 (8.3)	1,778 (8.0)	1,911 (8.5)
Heart Disease	25,513 (56.4)	1,445 (17.7)	1,327 (9.3)	2,772 (12.3)	300 (1.9)	659 (3.0)	1,048 (4.7)
Unknown	2,500 (5.5)	919 (11.2)	1,087 (7.6)	2,006 (8.9)	1,295 (8.1)	1,758 (7.9)	1,854 (8.3)
Stroke	4,478 (9.9)	4,854 (59.4)	7,736 (54.2)	12,590 (56.1)	184 (1.2)	336 (1.5)	290 (1.3)
Unknown	3,028 (6.7)	495 (6.1)	669 (4.7)	1,164 (5.2)	1,244 (7.8)	1,615 (7.3)	1,652 (7.4)
Cancer	240 (0.5)	48 (0.6)	92 (0.6)	140 (0.6)	57 (0.4)	18,378 (82.8)	217 (1.0)
Unknown	3,223 (7.1)	817 (10.0)	969 (6.8)	1,786 (8.0)	1,205 (7.6)	593 (2.7)	1,688 (7.6)
Asthma	2,619 (5.8)	540 (6.6)	743 (5.2)	1,283 (5.7)	289 (1.8)	1,176 (5.3)	18,372 (82.2)
Unknown	3,149 (7.0)	804 (9.8)	945 (6.6)	1,749 (7.8)	1,206 (7.6)	1,611 (7.3)	609 (2.7)
Medications (2004–14 only)							
Taking medications	8,819 (23.2)	1,339 (19.6)	3,881 (34.5)	5,220 (28.9)	672 (5.1)	7,193 (39.5)	5,923 (34.3)
Unknown	6,562 (17.3)	1,404 (20.5)	1,659 (14.8)	3,063 (16.9)	1,997 (15.1)	2,918 (16.0)	2,989 (17.3)
Missing	3,773 (9.9)	608 (8.9)	1,268 (11.3)	1,876 (10.4)	1,440 (10.9)	1,927 (10.6)	1,766 (10.2)
Smoking (men only)							
None	17,569 (55.4)	2,155 (48.1)	5,103 (58.2)	7,258 (54.8)	7,047 (57.9)	5,551 (45.1)	5,904 (44.1)
Cigarettes only	2,436 (7.7)	334 (7.5)	379 (4.3)	713 (5.4)	752 (6.2)	996 (8.1)	474 (3.5)
Bidis only	8,231 (26.0)	1,341 (29.9)	2,535 (28.9)	3,876 (29.3)	2,961 (24.3)	3,948 (32.1)	5,333 (39.9)
Cigarettes and bidis	2,477 (7.8)	538 (12.0)	449 (5.1)	987 (7.5)	932 (7.7)	1,446 (11.8)	1,199 (9.0)
Missing	999 (3.2)	114 (2.5)	296 (3.4)	410 (3.1)	486 (4.0)	360 (2.9)	465 (3.5)
Alcohol Use (men only)							
None	21,565 (68.0)	3,281 (73.2)	5,958 (68.0)	9,239 (69.8)	7,788 (64.0)	7,950 (64.6)	9,382 (70.1)
1–4	5,100 (16.1)	523 (11.7)	1,471 (16.8)	1,994 (15.1)	2,090 (17.2)	1,932 (15.7)	2,036 (15.2)
5–7	3,117 (9.8)	412 (9.2)	855 (9.8)	1,267 (9.6)	1,436 (11.8)	1,596 (13.0)	1,097 (8.2)
Unknown	584 (1.8)	97 (2.2)	132 (1.5)	229 (1.7)	272 (2.2)	306 (2.5)	237 (1.8)
Missing	1,346 (4.2)	169 (3.8)	346 (3.9)	515 (3.9)	592 (4.9)	517 (4.2)	623 (4.7)

eFigure 1 (next page) Total and stratified estimates of the association between (A) hypertension or (B) diabetes and ischemic heart disease mortality, displayed separately for each control group. Estimates are adjusted for age, sex, rurality, region, smoking, alcohol use, and year. The area of each shape is proportional to the sample size (cases and controls).

A. Hypertension and Ischemic Heart Disease

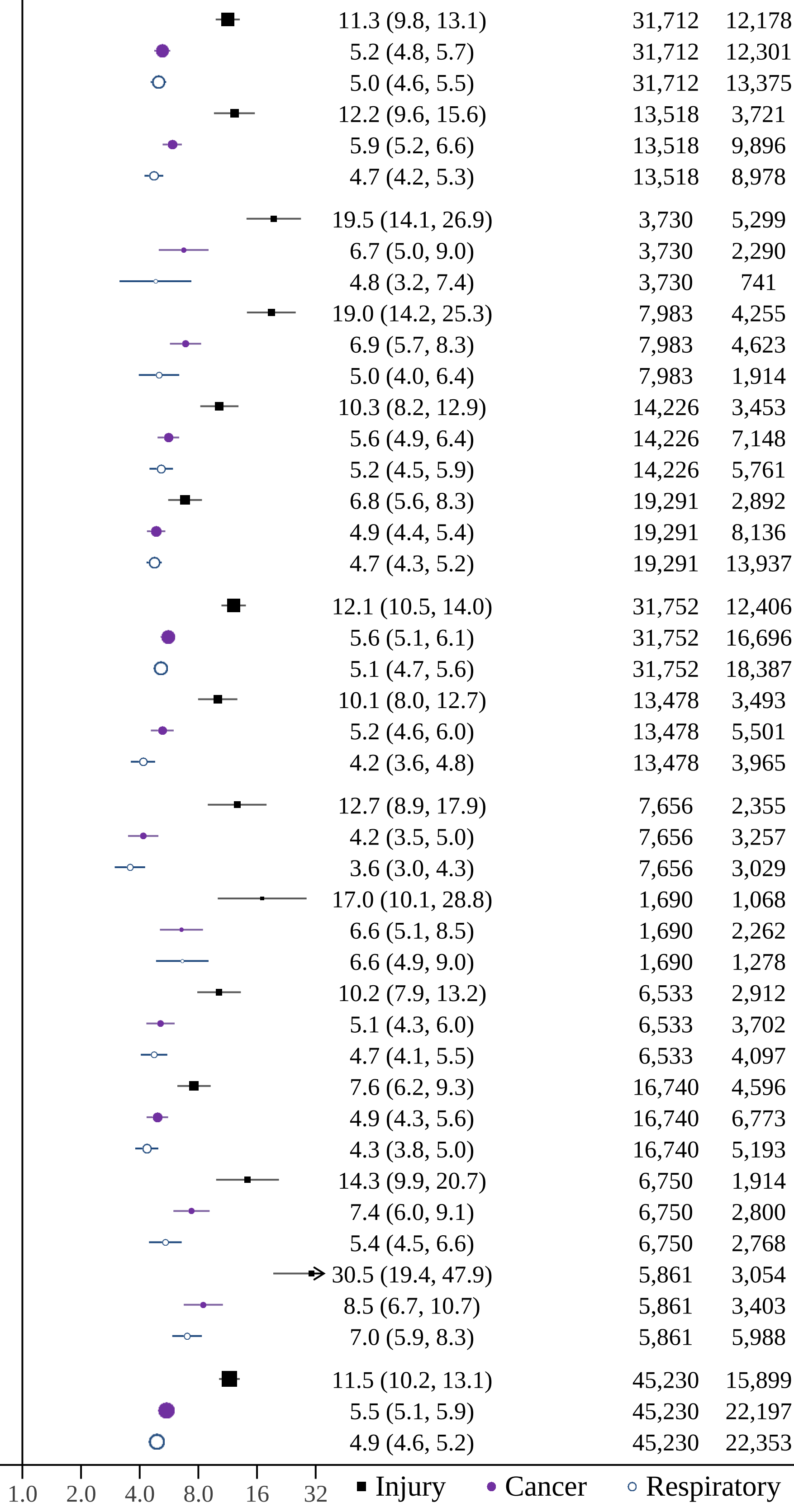

Group		Odds Ratio (99% confidence interval)	Cases	Controls
Sex	Male	11.3 (9.8, 13.1)	31,712	12,178
		5.2 (4.8, 5.7)	31,712	12,301
		5.0 (4.6, 5.5)	31,712	13,375
	Female	12.2 (9.6, 15.6)	13,518	3,721
		5.9 (5.2, 6.6)	13,518	9,896
		4.7 (4.2, 5.3)	13,518	8,978
Age (years)	30–39	19.5 (14.1, 26.9)	3,730	5,299
		6.7 (5.0, 9.0)	3,730	2,290
		4.8 (3.2, 7.4)	3,730	741
	40–49	19.0 (14.2, 25.3)	7,983	4,255
		6.9 (5.7, 8.3)	7,983	4,623
		5.0 (4.0, 6.4)	7,983	1,914
	50–59	10.3 (8.2, 12.9)	14,226	3,453
		5.6 (4.9, 6.4)	14,226	7,148
		5.2 (4.5, 5.9)	14,226	5,761
	60–69	6.8 (5.6, 8.3)	19,291	2,892
		4.9 (4.4, 5.4)	19,291	8,136
		4.7 (4.3, 5.2)	19,291	13,937
Residence	Rural	12.1 (10.5, 14.0)	31,752	12,406
		5.6 (5.1, 6.1)	31,752	16,696
		5.1 (4.7, 5.6)	31,752	18,387
	Urban	10.1 (8.0, 12.7)	13,478	3,493
		5.2 (4.6, 6.0)	13,478	5,501
		4.2 (3.6, 4.8)	13,478	3,965
Region	North	12.7 (8.9, 17.9)	7,656	2,355
		4.2 (3.5, 5.0)	7,656	3,257
		3.6 (3.0, 4.3)	7,656	3,029
	Northeast	17.0 (10.1, 28.8)	1,690	1,068
		6.6 (5.1, 8.5)	1,690	2,262
		6.6 (4.9, 9.0)	1,690	1,278
	East	10.2 (7.9, 13.2)	6,533	2,912
		5.1 (4.3, 6.0)	6,533	3,702
		4.7 (4.1, 5.5)	6,533	4,097
	South	7.6 (6.2, 9.3)	16,740	4,596
		4.9 (4.3, 5.6)	16,740	6,773
		4.3 (3.8, 5.0)	16,740	5,193
	West	14.3 (9.9, 20.7)	6,750	1,914
		7.4 (6.0, 9.1)	6,750	2,800
		5.4 (4.5, 6.6)	6,750	2,768
	Central	30.5 (19.4, 47.9)	5,861	3,054
		8.5 (6.7, 10.7)	5,861	3,403
		7.0 (5.9, 8.3)	5,861	5,988
	Total	11.5 (10.2, 13.1)	45,230	15,899
		5.5 (5.1, 5.9)	45,230	22,197
		4.9 (4.6, 5.2)	45,230	22,353

B. Diabetes and Ischemic Heart Disease

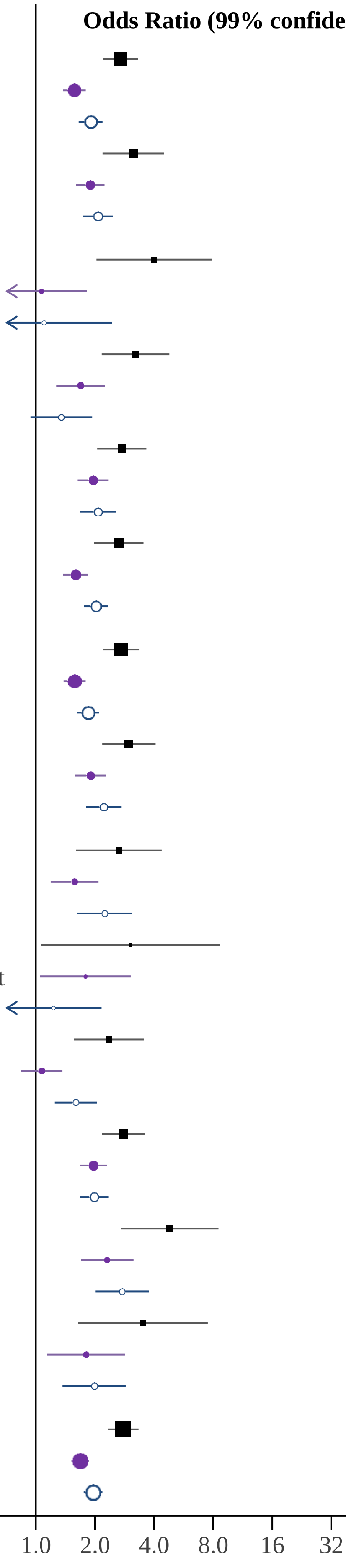

Group		Odds Ratio (99% confidence interval)	Cases	Controls
Sex	Male	2.7 (2.2, 3.3)	31,712	12,178
		1.6 (1.4, 1.8)	31,712	12,301
		1.9 (1.7, 2.2)	31,712	13,375
	Female	3.1 (2.2, 4.5)	13,518	3,721
		1.9 (1.6, 2.2)	13,518	9,896
		2.1 (1.7, 2.5)	13,518	8,978
Age (years)	30–39	4.0 (2.0, 7.9)	3,730	5,299
		1.1 (0.6, 1.8)	3,730	2,290
		1.1 (0.5, 2.4)	3,730	741
	40–49	3.2 (2.2, 4.8)	7,983	4,255
		1.7 (1.3, 2.3)	7,983	4,623
		1.3 (0.9, 1.9)	7,983	1,914
	50–59	2.7 (2.1, 3.7)	14,226	3,453
		2.0 (1.6, 2.4)	14,226	7,148
		2.1 (1.7, 2.6)	14,226	5,761
	60–69	2.6 (2.0, 3.5)	19,291	2,892
		1.6 (1.4, 1.9)	19,291	8,136
		2.0 (1.8, 2.3)	19,291	13,937
Residence	Rural	2.7 (2.2, 3.4)	31,752	12,406
		1.6 (1.4, 1.8)	31,752	16,696
		1.8 (1.6, 2.1)	31,752	18,387
	Urban	3.0 (2.2, 4.1)	13,478	3,493
		1.9 (1.6, 2.3)	13,478	5,501
		2.2 (1.8, 2.7)	13,478	3,965
Region	North	2.7 (1.6, 4.4)	7,656	2,355
		1.6 (1.2, 2.1)	7,656	3,257
		2.2 (1.6, 3.1)	7,656	3,029
	Northeast	3.0 (1.1, 8.7)	1,690	1,068
		1.8 (1.1, 3.0)	1,690	2,262
		1.2 (0.7, 2.2)	1,690	1,278
	East	2.4 (1.6, 3.5)	6,533	2,912
		1.1 (0.8, 1.4)	6,533	3,702
		1.6 (1.2, 2.0)	6,533	4,097
	South	2.8 (2.2, 3.6)	16,740	4,596
		2.0 (1.7, 2.3)	16,740	6,773
		2.0 (1.7, 2.4)	16,740	5,193
	West	4.8 (2.7, 8.5)	6,750	1,914
		2.3 (1.7, 3.1)	6,750	2,800
		2.8 (2.0, 3.8)	6,750	2,768
	Central	3.5 (1.6, 7.5)	5,861	3,054
		1.8 (1.1, 2.8)	5,861	3,403
		2.0 (1.4, 2.9)	5,861	5,988
	Total	2.8 (2.3, 3.3)	45,230	15,899
		1.7 (1.5, 1.9)	45,230	22,197
		2.0 (1.8, 2.2)	45,230	22,353

eFigure 2 Total and stratified estimates of the association between (A) hypertension or (B) diabetes and stroke mortality in high-burden states, displayed separately for each control group. Estimates are adjusted for age, sex, rurality, region, smoking, alcohol use, and year. The area of each shape is proportional to the sample size (cases and controls).

A. Hypertension and Stroke, High-Burden States

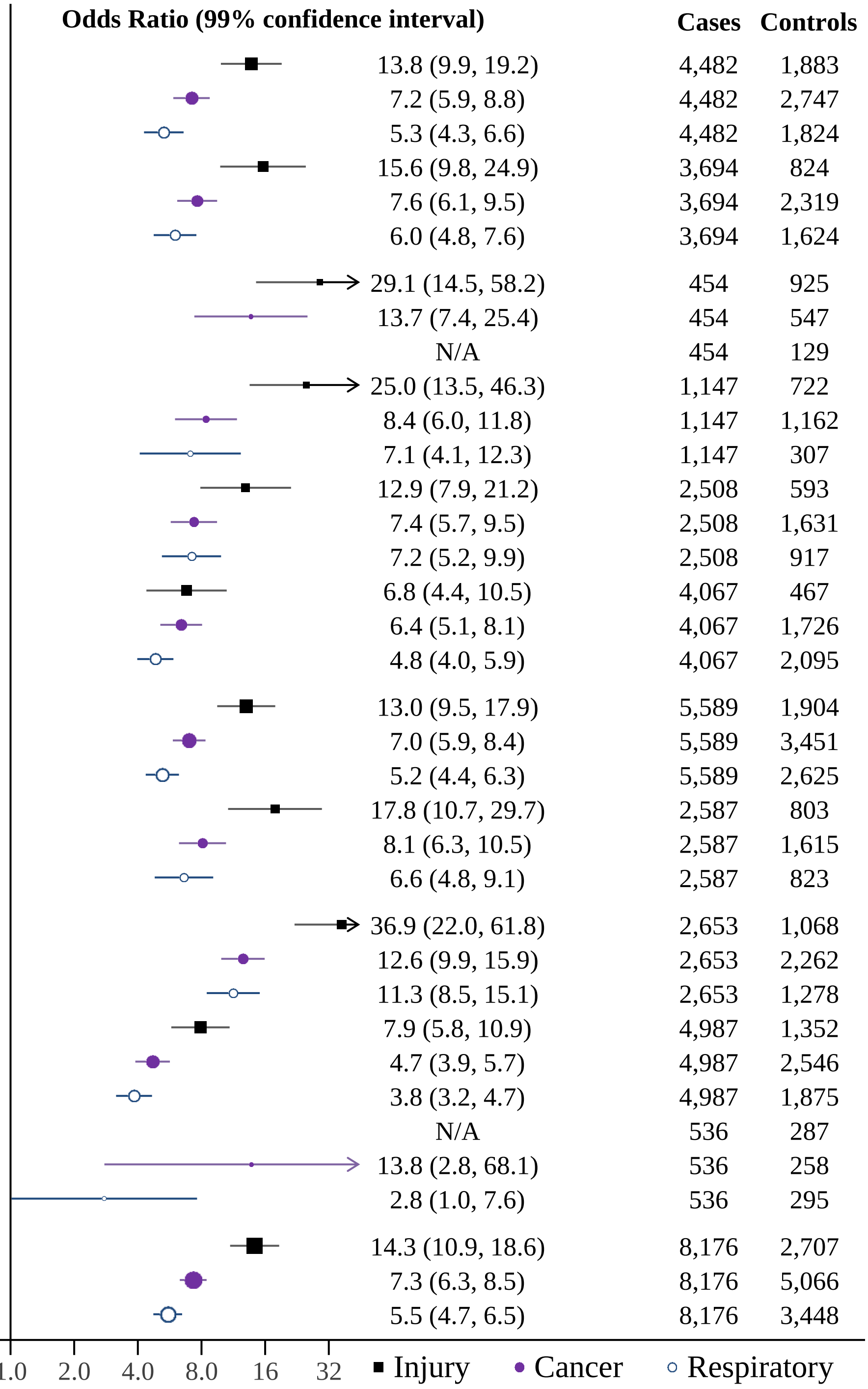

Group		Odds Ratio (99% confidence interval)	Cases	Controls
Sex	Male	13.8 (9.9, 19.2)	4,482	1,883
		7.2 (5.9, 8.8)	4,482	2,747
		5.3 (4.3, 6.6)	4,482	1,824
	Female	15.6 (9.8, 24.9)	3,694	824
		7.6 (6.1, 9.5)	3,694	2,319
		6.0 (4.8, 7.6)	3,694	1,624
Age (years)	30–39	29.1 (14.5, 58.2)	454	925
		13.7 (7.4, 25.4)	454	547
		N/A	454	129
	40–49	25.0 (13.5, 46.3)	1,147	722
		8.4 (6.0, 11.8)	1,147	1,162
		7.1 (4.1, 12.3)	1,147	307
	50–59	12.9 (7.9, 21.2)	2,508	593
		7.4 (5.7, 9.5)	2,508	1,631
		7.2 (5.2, 9.9)	2,508	917
	60–69	6.8 (4.4, 10.5)	4,067	467
		6.4 (5.1, 8.1)	4,067	1,726
		4.8 (4.0, 5.9)	4,067	2,095
Residence	Rural	13.0 (9.5, 17.9)	5,589	1,904
		7.0 (5.9, 8.4)	5,589	3,451
		5.2 (4.4, 6.3)	5,589	2,625
	Urban	17.8 (10.7, 29.7)	2,587	803
		8.1 (6.3, 10.5)	2,587	1,615
		6.6 (4.8, 9.1)	2,587	823
Region	Northeast	36.9 (22.0, 61.8)	2,653	1,068
		12.6 (9.9, 15.9)	2,653	2,262
		11.3 (8.5, 15.1)	2,653	1,278
	East	7.9 (5.8, 10.9)	4,987	1,352
		4.7 (3.9, 5.7)	4,987	2,546
		3.8 (3.2, 4.7)	4,987	1,875
	Central	N/A	536	287
		13.8 (2.8, 68.1)	536	258
		2.8 (1.0, 7.6)	536	295
	Total	14.3 (10.9, 18.6)	8,176	2,707
		7.3 (6.3, 8.5)	8,176	5,066
		5.5 (4.7, 6.5)	8,176	3,448

B. Diabetes and Stroke, High-Burden States

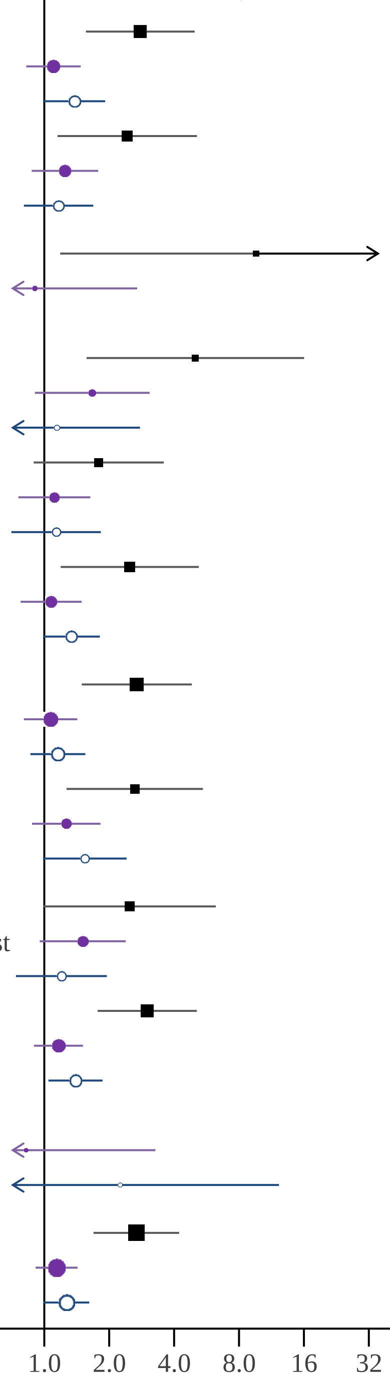

Group		Odds Ratio (99% confidence interval)	Cases	Controls
Sex	Male	2.8 (1.6, 5.0)	4,482	1,883
		1.1 (0.8, 1.5)	4,482	2,747
		1.4 (1.0, 1.9)	4,482	1,824
	Female	2.4 (1.2, 5.1)	3,694	824
		1.2 (0.9, 1.8)	3,694	2,319
		1.2 (0.8, 1.7)	3,694	1,624
Age (years)	30–39	9.6 (1.2, 77.8)	454	925
		0.9 (0.3, 2.7)	454	547
		N/A	454	129
	40–49	5.0 (1.6, 16.0)	1,147	722
		1.7 (0.9, 3.1)	1,147	1,162
		1.1 (0.5, 2.8)	1,147	307
	50–59	1.8 (0.9, 3.6)	2,508	593
		1.1 (0.8, 1.6)	2,508	1,631
		1.1 (0.7, 1.8)	2,508	917
	60–69	2.5 (1.2, 5.2)	4,067	467
		1.1 (0.8, 1.5)	4,067	1,726
		1.3 (1.0, 1.8)	4,067	2,095
Residence	Rural	2.7 (1.5, 4.8)	5,589	1,904
		1.1 (0.8, 1.4)	5,589	3,451
		1.2 (0.9, 1.6)	5,589	2,625
	Urban	2.6 (1.3, 5.4)	2,587	803
		1.3 (0.9, 1.8)	2,587	1,615
		1.5 (1.0, 2.4)	2,587	823
Region	Northeast	2.5 (1.0, 6.2)	2,653	1,068
		1.5 (1.0, 2.4)	2,653	2,262
		1.2 (0.7, 1.9)	2,653	1,278
	East	3.0 (1.8, 5.1)	4,987	1,352
		1.2 (0.9, 1.5)	4,987	2,546
		1.4 (1.0, 1.9)	4,987	1,875
	Central	N/A	536	287
		0.8 (0.2, 3.3)	536	258
		2.2 (0.4, 12.3)	536	295
	Total	2.7 (1.7, 4.2)	8,176	2,707
		1.1 (0.9, 1.4)	8,176	5,066
		1.3 (1.0, 1.6)	8,176	3,448

■ Injury ● Cancer ○ Respiratory

eFigure 3 (next page) Total and stratified estimates of the association between (A) hypertension or (B) diabetes and stroke mortality in low-burden states, displayed separately for each control group. Estimates are adjusted for age, sex, rurality, region, smoking, alcohol use, and year. The area of each shape is proportional to the sample size (cases and controls).

A. Hypertension and Stroke, Low-Burden States

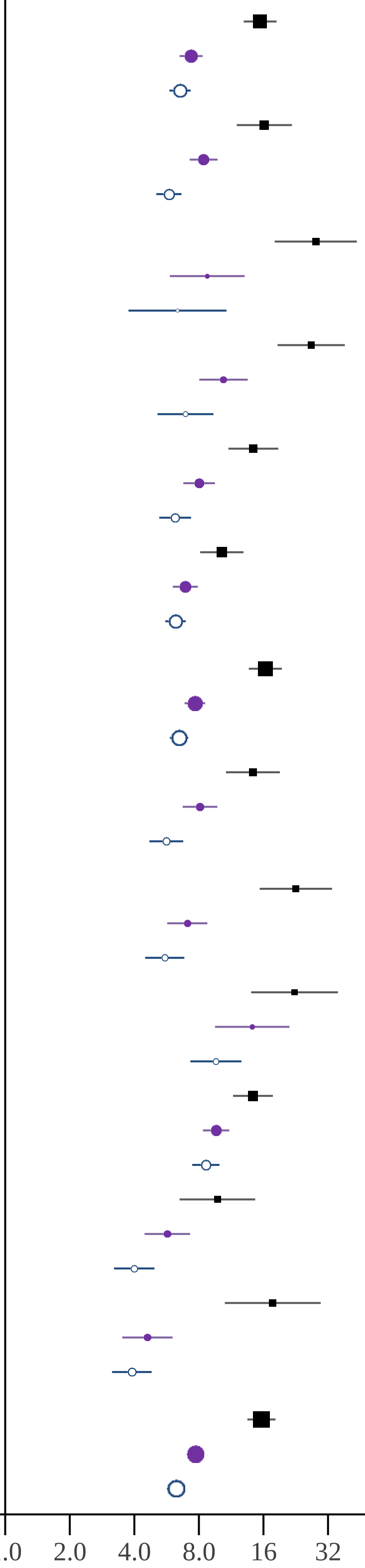

Group		Odds Ratio (99% confidence interval)	Cases	Controls
Sex	Male	15.4 (12.9, 18.4)	8,762	10,295
		7.4 (6.5, 8.3)	8,762	9,554
		6.5 (5.8, 7.3)	8,762	11,551
	Female	16.2 (12.0, 21.7)	5,513	2,897
		8.4 (7.2, 9.8)	5,513	7,577
		5.8 (5.1, 6.6)	5,513	7,354
Age (years)	30–39	28.1 (18.1, 43.6)	630	4,374
		8.8 (5.9, 13.1)	630	1,743
		6.4 (3.8, 10.8)	630	612
	40–49	26.7 (18.6, 38.3)	1,533	3,533
		10.4 (8.0, 13.5)	1,533	3,461
		6.9 (5.1, 9.4)	1,533	1,607
	50–59	14.4 (11.0, 18.8)	3,918	2,860
		8.0 (6.8, 9.5)	3,918	5,517
		6.2 (5.2, 7.4)	3,918	4,844
	60–69	10.2 (8.1, 12.9)	8,194	2,425
		6.9 (6.0, 7.9)	8,194	6,410
		6.2 (5.6, 6.9)	8,194	11,842
Residence	Rural	16.3 (13.7, 19.5)	11,101	10,502
		7.7 (6.9, 8.6)	11,101	13,245
		6.5 (5.9, 7.1)	11,101	15,762
	Urban	14.3 (10.7, 19.1)	3,174	2,690
		8.1 (6.7, 9.8)	3,174	3,886
		5.6 (4.7, 6.8)	3,174	3,142
Region	North	22.7 (15.4, 33.4)	1,861	2,355
		7.1 (5.7, 8.8)	1,861	3,257
		5.5 (4.5, 6.8)	1,861	3,029
	East	22.3 (14.0, 35.6)	1,936	1,560
		14.2 (9.5, 21.2)	1,936	1,156
		9.6 (7.3, 12.6)	1,936	2,222
	South	14.3 (11.5, 17.7)	5,633	4,596
		9.6 (8.3, 11.1)	5,633	6,773
		8.6 (7.4, 10.0)	5,633	5,193
	West	9.8 (6.5, 14.6)	2,496	1,914
		5.7 (4.5, 7.3)	2,496	2,800
		4.0 (3.2, 5.0)	2,496	2,768
	Central	17.7 (10.6, 29.6)	2,349	2,767
		4.6 (3.5, 6.0)	2,349	3,145
		3.9 (3.1, 4.8)	2,349	5,693
	Total	15.7 (13.5, 18.2)	14,275	13,192
		7.7 (7.0, 8.5)	14,275	17,131
		6.3 (5.7, 6.8)	14,275	18,905

B. Diabetes and Stroke, Low-Burden States

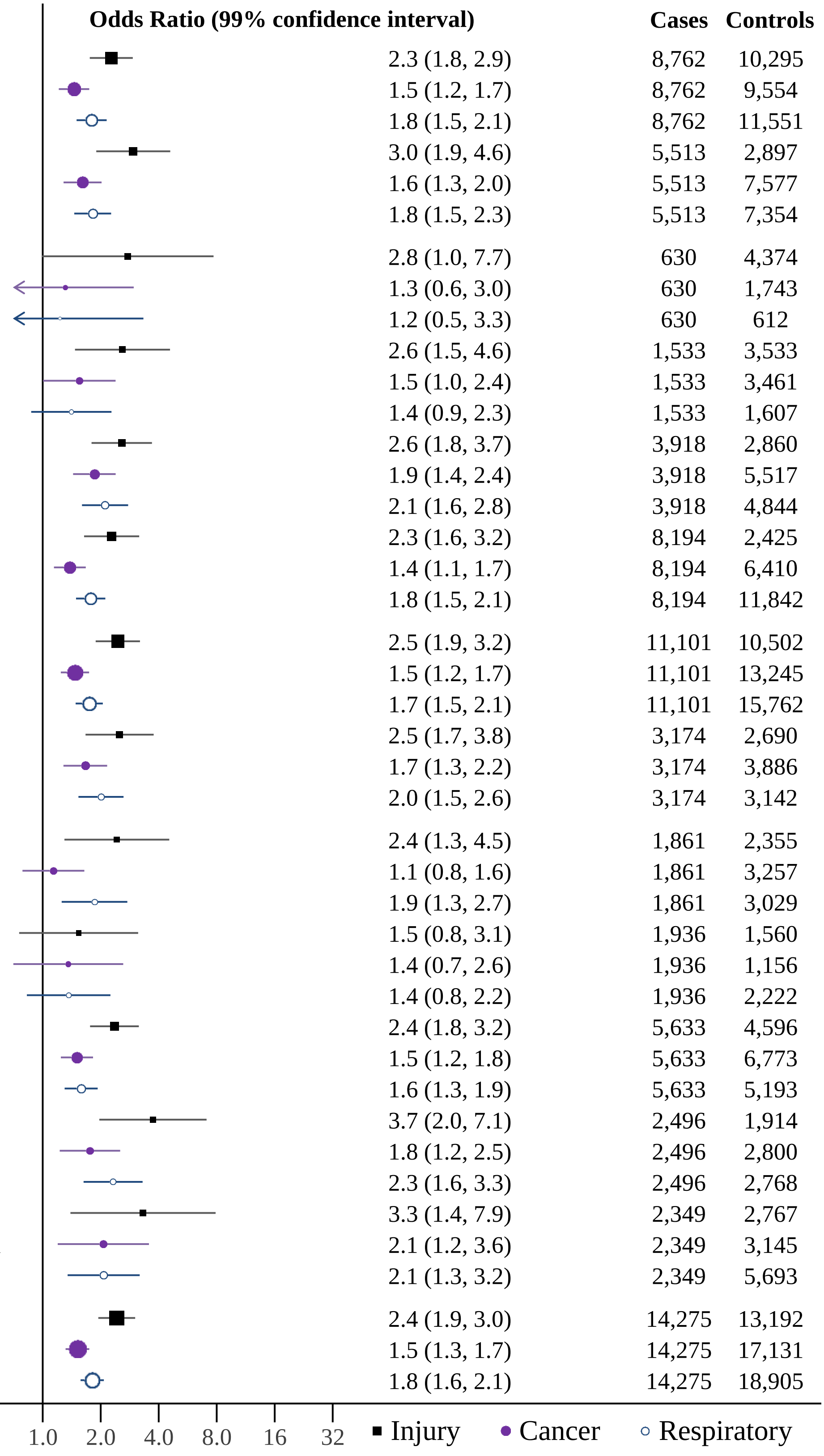

Group		Odds Ratio (99% confidence interval)	Cases	Controls
Sex	Male	2.3 (1.8, 2.9)	8,762	10,295
		1.5 (1.2, 1.7)	8,762	9,554
		1.8 (1.5, 2.1)	8,762	11,551
	Female	3.0 (1.9, 4.6)	5,513	2,897
		1.6 (1.3, 2.0)	5,513	7,577
		1.8 (1.5, 2.3)	5,513	7,354
Age (years)	30–39	2.8 (1.0, 7.7)	630	4,374
		1.3 (0.6, 3.0)	630	1,743
		1.2 (0.5, 3.3)	630	612
	40–49	2.6 (1.5, 4.6)	1,533	3,533
		1.5 (1.0, 2.4)	1,533	3,461
		1.4 (0.9, 2.3)	1,533	1,607
	50–59	2.6 (1.8, 3.7)	3,918	2,860
		1.9 (1.4, 2.4)	3,918	5,517
		2.1 (1.6, 2.8)	3,918	4,844
	60–69	2.3 (1.6, 3.2)	8,194	2,425
		1.4 (1.1, 1.7)	8,194	6,410
		1.8 (1.5, 2.1)	8,194	11,842
Residence	Rural	2.5 (1.9, 3.2)	11,101	10,502
		1.5 (1.2, 1.7)	11,101	13,245
		1.7 (1.5, 2.1)	11,101	15,762
	Urban	2.5 (1.7, 3.8)	3,174	2,690
		1.7 (1.3, 2.2)	3,174	3,886
		2.0 (1.5, 2.6)	3,174	3,142
Region	North	2.4 (1.3, 4.5)	1,861	2,355
		1.1 (0.8, 1.6)	1,861	3,257
		1.9 (1.3, 2.7)	1,861	3,029
	East	1.5 (0.8, 3.1)	1,936	1,560
		1.4 (0.7, 2.6)	1,936	1,156
		1.4 (0.8, 2.2)	1,936	2,222
	South	2.4 (1.8, 3.2)	5,633	4,596
		1.5 (1.2, 1.8)	5,633	6,773
		1.6 (1.3, 1.9)	5,633	5,193
	West	3.7 (2.0, 7.1)	2,496	1,914
		1.8 (1.2, 2.5)	2,496	2,800
		2.3 (1.6, 3.3)	2,496	2,768
	Central	3.3 (1.4, 7.9)	2,349	2,767
		2.1 (1.2, 3.6)	2,349	3,145
		2.1 (1.3, 3.2)	2,349	5,693
	Total	2.4 (1.9, 3.0)	14,275	13,192
		1.5 (1.3, 1.7)	14,275	17,131
		1.8 (1.6, 2.1)	14,275	18,905

eFigure 4 (next page) Association between hypertension (A) or diabetes (B) and stroke mortality in high-burden states. The total and stratified estimates are adjusted for age, sex, urban/rural residence, region, smoking, alcohol use, and year. The area of each box is proportional to the sample size (cases and controls).

A. Hypertension and stroke, high-burden states

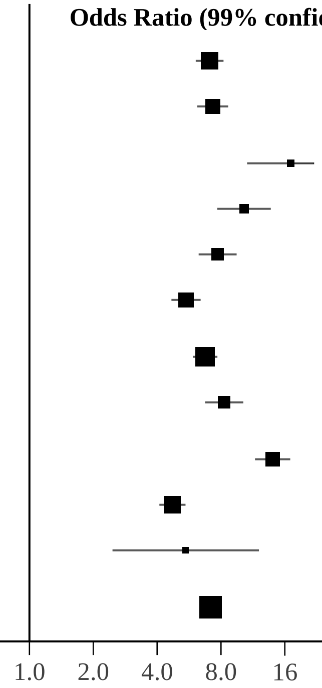

Group		Odds Ratio (99% confidence interval)	Cases	Controls
Sex	Male	7.1 (6.1, 8.2)	4,482	6,454
	Female	7.3 (6.2, 8.7)	3,694	4,767
Age (years)	30–39	17.0 (10.6, 27.4)	454	1,601
	40–49	10.3 (7.7, 13.7)	1,147	2,191
	50–59	7.7 (6.3, 9.5)	2,508	3,141
	60–69	5.5 (4.7, 6.4)	4,067	4,288
Residence	Rural	6.7 (5.9, 7.7)	5,589	7,980
	Urban	8.3 (6.7, 10.2)	2,587	3,241
Region	Northeast	14.0 (11.6, 17.0)	2,653	4,608
	East	4.7 (4.1, 5.4)	4,987	5,773
	Central	5.5 (2.5, 12.1)	536	840
Total		7.1 (6.4, 8.0)	8,176	11,221

B. Diabetes and stroke, high-burden states

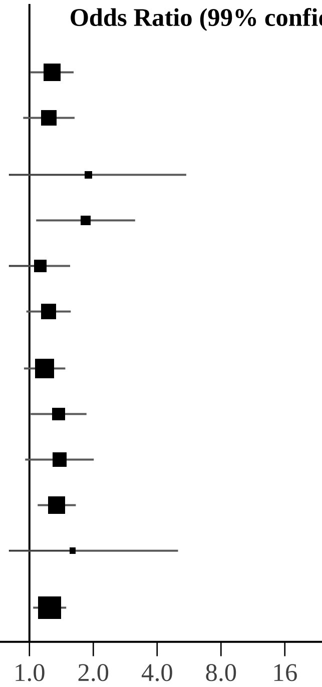

Group		Odds Ratio (99% confidence interval)	Cases	Controls
Sex	Male	1.3 (1.0, 1.6)	4,482	6,454
	Female	1.2 (0.9, 1.6)	3,694	4,767
Age (years)	30–39	1.9 (0.7, 5.5)	454	1,601
	40–49	1.8 (1.1, 3.1)	1,147	2,191
	50–59	1.1 (0.8, 1.6)	2,508	3,141
	60–69	1.2 (1.0, 1.6)	4,067	4,288
Residence	Rural	1.2 (0.9, 1.5)	5,589	7,980
	Urban	1.4 (1.0, 1.9)	2,587	3,241
	Northeast	1.4 (1.0, 2.0)	2,653	4,608
	East	1.3 (1.1, 1.7)	4,987	5,773
	Central	1.6 (0.5, 5.0)	536	840
Total		1.2 (1.0, 1.5)	8,176	11,221

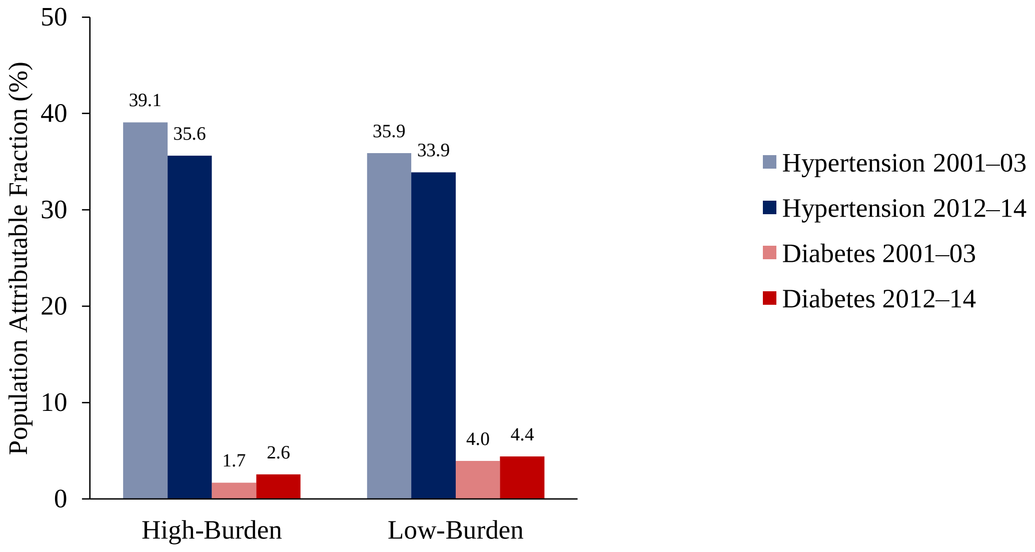

eFigure 5 Population attributable fractions (%) for stroke in high- and low-burden states during the beginning (2001–03) and end (2012–14) of the study period. Blue bars represent hypertension; red bars represent diabetes.

eFigure 6 (next page) Total and stratified estimates of the association between hypertension, diabetes, or both and (A) ischemic heart disease, (B) stroke (high-burden states), and (C) stroke (low-burden states). Estimates are adjusted for age, sex, rurality, region, smoking, alcohol use, and year. The area of each box is proportional to the sample size (cases and controls).

A. Ischemic Heart Disease

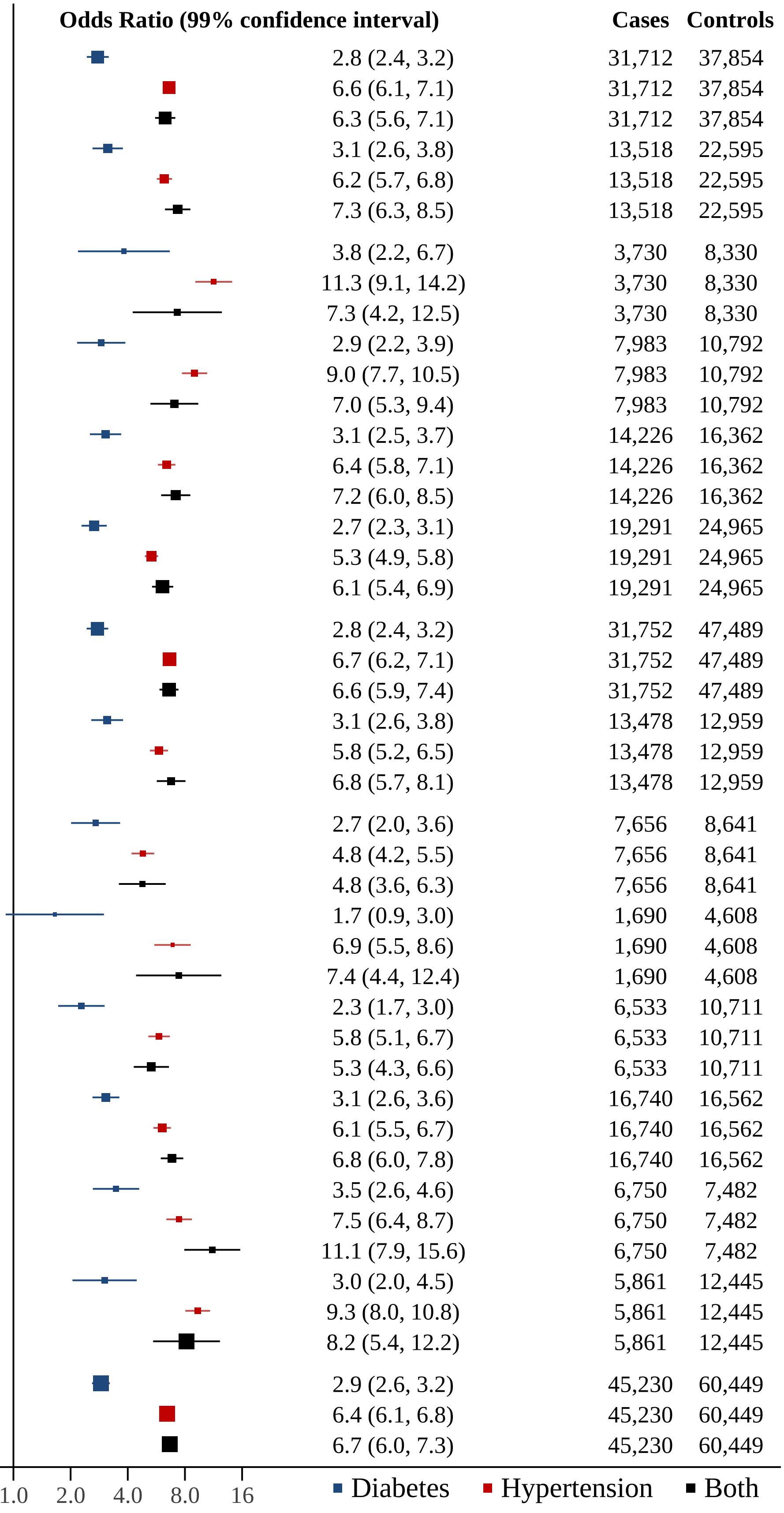

Group		Odds Ratio (99% confidence interval)	Cases	Controls
Sex	Male	2.8 (2.4, 3.2)	31,712	37,854
		6.6 (6.1, 7.1)	31,712	37,854
		6.3 (5.6, 7.1)	31,712	37,854
	Female	3.1 (2.6, 3.8)	13,518	22,595
		6.2 (5.7, 6.8)	13,518	22,595
		7.3 (6.3, 8.5)	13,518	22,595
Age (years)	30–39	3.8 (2.2, 6.7)	3,730	8,330
		11.3 (9.1, 14.2)	3,730	8,330
		7.3 (4.2, 12.5)	3,730	8,330
	40–49	2.9 (2.2, 3.9)	7,983	10,792
		9.0 (7.7, 10.5)	7,983	10,792
		7.0 (5.3, 9.4)	7,983	10,792
	50–59	3.1 (2.5, 3.7)	14,226	16,362
		6.4 (5.8, 7.1)	14,226	16,362
		7.2 (6.0, 8.5)	14,226	16,362
	60–69	2.7 (2.3, 3.1)	19,291	24,965
		5.3 (4.9, 5.8)	19,291	24,965
		6.1 (5.4, 6.9)	19,291	24,965
Residence	Rural	2.8 (2.4, 3.2)	31,752	47,489
		6.7 (6.2, 7.1)	31,752	47,489
		6.6 (5.9, 7.4)	31,752	47,489
	Urban	3.1 (2.6, 3.8)	13,478	12,959
		5.8 (5.2, 6.5)	13,478	12,959
		6.8 (5.7, 8.1)	13,478	12,959
Region	North	2.7 (2.0, 3.6)	7,656	8,641
		4.8 (4.2, 5.5)	7,656	8,641
		4.8 (3.6, 6.3)	7,656	8,641
	Northeast	1.7 (0.9, 3.0)	1,690	4,608
		6.9 (5.5, 8.6)	1,690	4,608
		7.4 (4.4, 12.4)	1,690	4,608
	East	2.3 (1.7, 3.0)	6,533	10,711
		5.8 (5.1, 6.7)	6,533	10,711
		5.3 (4.3, 6.6)	6,533	10,711
	South	3.1 (2.6, 3.6)	16,740	16,562
		6.1 (5.5, 6.7)	16,740	16,562
		6.8 (6.0, 7.8)	16,740	16,562
	West	3.5 (2.6, 4.6)	6,750	7,482
		7.5 (6.4, 8.7)	6,750	7,482
		11.1 (7.9, 15.6)	6,750	7,482
	Central	3.0 (2.0, 4.5)	5,861	12,445
		9.3 (8.0, 10.8)	5,861	12,445
		8.2 (5.4, 12.2)	5,861	12,445
	Total	2.9 (2.6, 3.2)	45,230	60,449
		6.4 (6.1, 6.8)	45,230	60,449
		6.7 (6.0, 7.3)	45,230	60,449

B. Stroke, High-Burden States

Group		Odds Ratio (99% confidence interval)	Cases	Controls
		1.9 (1.3, 2.7)	4,482	6,454
	Male	6.8 (5.8, 8.0)	4,482	6,454
		5.9 (4.5, 7.7)	4,482	6,454
Sex		2.0 (1.3, 3.2)	3,694	4,767
	Female	6.9 (5.8, 8.3)	3,694	4,767
		5.7 (4.1, 7.8)	3,694	4,767
		3.7 (0.7, 18.2)	454	1,601
	30–39	15.3 (9.4, 25.0)	454	1,601
		18.0 (5.4, 60.4)	454	1,601
		2.9 (1.4, 6.2)	1,147	2,191
	40–49	9.8 (7.3, 13.3)	1,147	2,191
		9.9 (5.2, 18.9)	1,147	2,191
Age (years)		1.9 (1.2, 3.1)	2,508	3,141
	50–59	7.3 (5.8, 9.1)	2,508	3,141
		5.4 (3.7, 7.8)	2,508	3,141
		1.7 (1.2, 2.5)	4,067	4,288
	60–69	5.2 (4.4, 6.2)	4,067	4,288
		4.8 (3.7, 6.4)	4,067	4,288
		1.7 (1.2, 2.3)	5,589	7,980
	Rural	6.4 (5.5, 7.4)	5,589	7,980
		5.5 (4.2, 7.1)	5,589	7,980
Residence		2.6 (1.6, 4.1)	2,587	3,241
	Urban	8.1 (6.5, 10.1)	2,587	3,241
		6.3 (4.5, 8.8)	2,587	3,241
		2.0 (1.2, 3.4)	2,653	4,608
	Northeast	12.6 (10.3, 15.4)	2,653	4,608
		12.1 (7.6, 19.4)	2,653	4,608
		1.9 (1.4, 2.6)	4,987	5,773
	East	4.5 (3.9, 5.2)	4,987	5,773
Region		4.4 (3.5, 5.6)	4,987	5,773
		1.7 (0.4, 6.9)	536	840
	Central	5.6 (2.5, 12.7)	536	840
		5.6 (1.0, 32.7)	536	840
		1.9 (1.5, 2.5)	8,176	11,221
	Total	6.8 (6.1, 7.7)	8,176	11,221
		5.7 (4.6, 7.0)	8,176	11,221

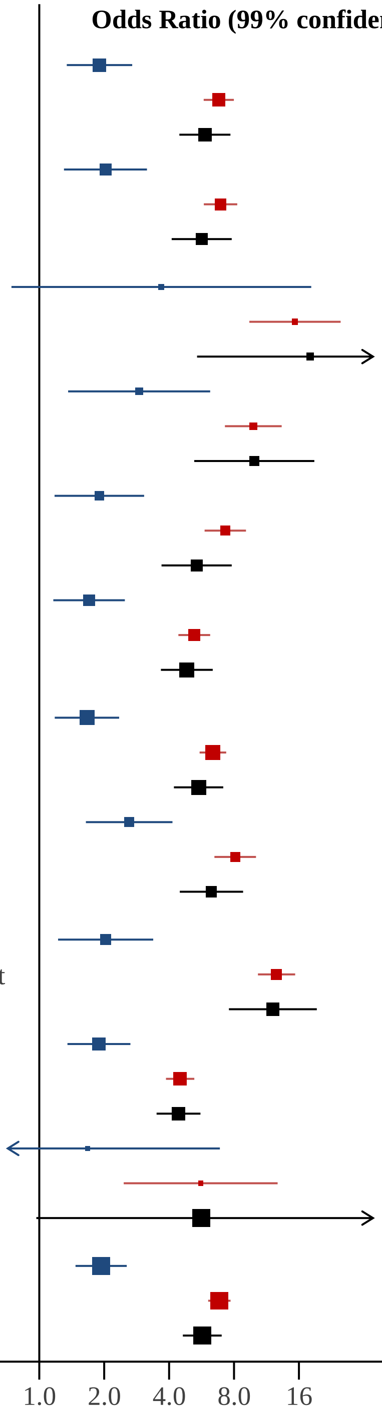

■ Diabetes ■ Hypertension ■ Both

C. Stroke, Low-Burden States

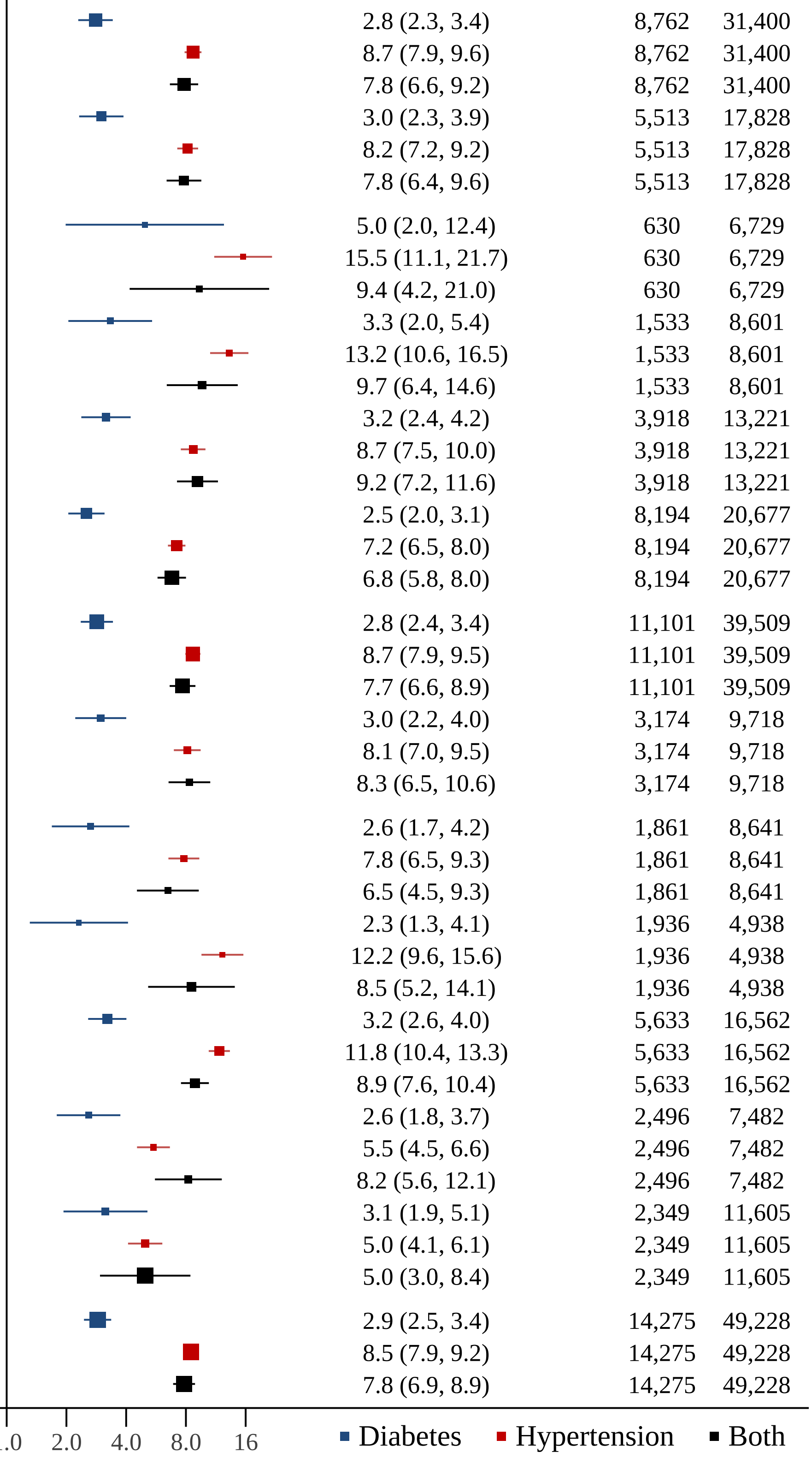

Group		Odds Ratio (99% confidence interval)	Cases	Controls
Sex	Male	2.8 (2.3, 3.4)	8,762	31,400
		8.7 (7.9, 9.6)	8,762	31,400
		7.8 (6.6, 9.2)	8,762	31,400
	Female	3.0 (2.3, 3.9)	5,513	17,828
		8.2 (7.2, 9.2)	5,513	17,828
		7.8 (6.4, 9.6)	5,513	17,828
Age (years)	30–39	5.0 (2.0, 12.4)	630	6,729
		15.5 (11.1, 21.7)	630	6,729
		9.4 (4.2, 21.0)	630	6,729
	40–49	3.3 (2.0, 5.4)	1,533	8,601
		13.2 (10.6, 16.5)	1,533	8,601
		9.7 (6.4, 14.6)	1,533	8,601
	50–59	3.2 (2.4, 4.2)	3,918	13,221
		8.7 (7.5, 10.0)	3,918	13,221
		9.2 (7.2, 11.6)	3,918	13,221
	60–69	2.5 (2.0, 3.1)	8,194	20,677
		7.2 (6.5, 8.0)	8,194	20,677
		6.8 (5.8, 8.0)	8,194	20,677
Residence	Rural	2.8 (2.4, 3.4)	11,101	39,509
		8.7 (7.9, 9.5)	11,101	39,509
		7.7 (6.6, 8.9)	11,101	39,509
	Urban	3.0 (2.2, 4.0)	3,174	9,718
		8.1 (7.0, 9.5)	3,174	9,718
		8.3 (6.5, 10.6)	3,174	9,718
Region	North	2.6 (1.7, 4.2)	1,861	8,641
		7.8 (6.5, 9.3)	1,861	8,641
		6.5 (4.5, 9.3)	1,861	8,641
	East	2.3 (1.3, 4.1)	1,936	4,938
		12.2 (9.6, 15.6)	1,936	4,938
		8.5 (5.2, 14.1)	1,936	4,938
	South	3.2 (2.6, 4.0)	5,633	16,562
		11.8 (10.4, 13.3)	5,633	16,562
		8.9 (7.6, 10.4)	5,633	16,562
	West	2.6 (1.8, 3.7)	2,496	7,482
		5.5 (4.5, 6.6)	2,496	7,482
		8.2 (5.6, 12.1)	2,496	7,482
	Central	3.1 (1.9, 5.1)	2,349	11,605
		5.0 (4.1, 6.1)	2,349	11,605
		5.0 (3.0, 8.4)	2,349	11,605
	Total	2.9 (2.5, 3.4)	14,275	49,228
		8.5 (7.9, 9.2)	14,275	49,228
		7.8 (6.9, 8.9)	14,275	49,228

eTable 3 Association between hypertension and diabetes with ischemic heart disease and stroke by state. Some states located in the northeast region were combined due to low sample size. These states are s Sikkim, Arunachal Pradesh, Nagaland, Manipur, Mizoram, Tripura, and Meghalaya. High burden states for male stroke deaths were Assam, West Bengal, Chhattisgarh, and the northeast states. High burden states for female stroke deaths were Assam, West Bengal, Orissa, Chhattisgarh, and the northeastern states.

Outcome	Risk Factor	State	Odds Ratio (99% CI)	Cases	Controls
Ischemic Heart Disease	Hypertension	Bihar	12.0 (8.9–16.2)	1801	2759
		Uttar Pradesh	11.9 (9.5–14.8)	2091	5531
		Rajasthan	9.8 (7.6–12.6)	1510	3428
		Gujarat	9.6 (7.6–12.1)	2657	3278
		Orissa	9.3 (6.8–12.7)	1046	2331
		Madhya Pradesh	9.2 (6.2–13.7)	1642	2645
		Andhra Pradesh	8.5 (6.8–10.6)	4107	3851
		Chhattisgarh	8.2 (4.0–17.1)	618	840
		Northeast States	7.6 (5.8–10.0)	1028	2827
		Tamil Nadu	7.6 (6.2–9.3)	4989	3891
		Assam	7.5 (5.2–10.7)	662	1781
		Jharkhand	6.9 (4.3–11.1)	613	782
		Haryana	6.5 (4.3–9.9)	1709	1895
		Delhi	5.9 (3.6–9.5)	926	824
		Maharashtra	5.5 (4.4–6.9)	2960	3159
		Jammu & Kashmir	5.4 (4.1–7.2)	1104	1856
		Karnataka	3.9 (3.2–4.8)	3811	4617
		West Bengal	3.8 (3.2–4.4)	3073	4839
		Kerala	3.3 (2.8–4.0)	2939	3278
		Punjab	3.2 (2.5–4.0)	2458	1729
	Diabetes	Kerala	2.9 (2.3–3.5)	2939	3278
		Tamil Nadu	2.6 (2.0–3.4)	4989	3891
		Rajasthan	2.6 (1.3–5.1)	1510	3428
		Maharashtra	2.6 (1.8–3.7)	2960	3159
		Chhattisgarh	2.6 (0.9–7.6)	618	840
		Haryana	2.5 (1.3–4.9)	1709	1895
		Madhya Pradesh	2.4 (1.1–5.3)	1642	2645
		Gujarat	2.3 (1.5–3.4)	2657	3278
		Delhi	2.2 (1.2–4.0)	926	824
		Orissa	2.1 (1.2–3.6)	1046	2331
		Karnataka	1.9 (1.4–2.5)	3811	4617
		Assam	1.8 (0.9–3.3)	662	1781
		Bihar	1.6 (1.0–2.7)	1801	2759
		Jammu & Kashmir	1.6 (0.8–3.1)	1104	1856
		Uttar Pradesh	1.5 (0.9–2.3)	2091	5531
		Northeast States	1.4 (0.7–2.6)	1028	2827
		West Bengal	1.4 (1.1–1.7)	3073	4839
		Jharkhand	1.3 (0.6–3.1)	613	782
		Punjab	1.2 (0.9–1.8)	2458	1729
		Andhra Pradesh	0.9 (0.7–1.3)	4107	3851

Stroke, High Burden	Hypertension	Assam	23.6 (17.3–32.2)	1251	1781
		Northeast states (excluding Assam)	9.3 (7.2–11.9)	1402	2827
		West Bengal	4.1 (3.5–4.8)	4188	4839
		Chhattisgarh	5.5 (2.5–12.1)	536	840
		Orissa (women)		799	934
	Diabetes	Chhattisgarh	1.6 (0.5–5.0)	536	840
		Northeast states (excluding Assam)	1.5 (0.9–2.5)	1402	2827
		West Bengal	1.4 (1.1–1.7)	4188	4839
		Assam	1.2 (0.7–2.0)	1251	1781
		Orissa (women)		799	934
Stroke, Low Burden	Hypertension	Andhra Pradesh	29.2 (22.4–37.9)	1445	3851
		Kerala	17.6 (13.7–22.7)	1087	3278
		Bihar	13.1 (9.2–18.6)	839	2759
		Jammu & Kashmir	12.1 (8.2–18.0)	388	1856
		Jharkhand	11.0 (6.2–19.6)	254	782
		Haryana	9.7 (5.7–16.4)	359	1895
		Punjab	8.2 (5.8–11.6)	462	1729
		Gujara	2.6 (2.2–3.0)	737	3278
		Rajasthan	7.3 (5.2–10.2)	547	3428
		Delhi	5.6 (2.8–11.3)	226	824
		Karnataka	5.5 (4.4–6.9)	1560	4617
		Tamil Nadu	5.4 (4.2–7.0)	1193	3891
		Uttar Pradesh	5.3 (4.0–7.2)	887	5531
		Madhya Pradesh	4.8 (3.0–7.7)	915	2645
		Maharashtra	3.7 (2.8–4.8)	1326	3159
		Orissa (men)		843	1397
	Diabetes	Madhya Pradesh	3.6 (1.6–8.0)	915	2645
		Tamil Nadu	2.6 (1.9–3.7)	1193	3891
		Haryana	2.6 (1.0–7.0)	359	1895
		Maharashtra	2.3 (1.5–3.6)	1326	3159
		Rajasthan	1.7 (0.7–4.2)	547	3428
		Karnataka	1.7 (1.2–2.3)	1560	4617
		Uttar Pradesh	1.6 (1.0–2.8)	887	5531
		Gujarat	1.6 (0.8–3.4)	737	3278
		Delhi	1.5 (0.6–3.6)	226	824
		Kerala	1.3 (0.9–1.7)	1087	3278
		Jharkhand	1.2 (0.4–3.5)	254	782
		Jammu & Kashmir	1.2 (0.4–3.1)	388	1856
		Punjab	1.1 (0.7–1.9)	462	1729
		Andhra Pradesh	0.8 (0.5–1.2)	1445	3851
		Bihar	0.7 (0.4–1.5)	839	2759
		Orissa (men)		843	1397

*Odds ratios for stroke in Orissa could not be computed due to sample size limitations.

Abbreviations: CI, confidence interval

eFigure 7 (next page) Total and stratified estimates of the association between (A) hypertension or (B) diabetes and ischemic heart disease mortality, where both physicians initially agreed on cause of death category without further adjudication. Estimates are adjusted for age, sex, rurality, region, smoking, alcohol use, and year*. The area of each box is proportional to the sample size (cases and controls).

*We tested whether the effects of diabetes and hypertension varied by year. There was a significant interaction between diabetes and year (p=0.0001) and hypertension and year (p=0.0002). These interaction terms were included in all models.

A. Hypertension and Ischemic Heart Disease

Group		Odds Ratio (99% confidence interval)	Cases	Controls
Sex	Male	6.1 (5.7, 6.5)	26,890	37,854
	Female	5.8 (5.3, 6.4)	10,915	22,595
Age (years)	30–39	10.2 (8.2, 12.8)	3,098	8,330
	40–49	8.4 (7.3, 9.7)	6,935	10,792
	50–59	5.9 (5.4, 6.6)	12,154	16,362
	60–69	5.1 (4.7, 5.5)	15,618	24,965
Residence	Rural	6.3 (5.9, 6.7)	26,397	47,489
	Urban	5.3 (4.8, 5.9)	11,408	12,959
Region	North	4.5 (4.0, 5.2)	6,588	8,641
	Northeast	7.0 (5.5, 8.9)	1,109	4,608
	East	6.2 (5.4, 7.0)	4,768	10,711
	South	5.1 (4.6, 5.6)	14,771	16,562
	West	7.1 (6.1, 8.2)	5,763	7,482
	Central	10.0 (8.6, 11.6)	4,806	12,445
Total		6.0 (5.7, 6.3)	37,805	60,449

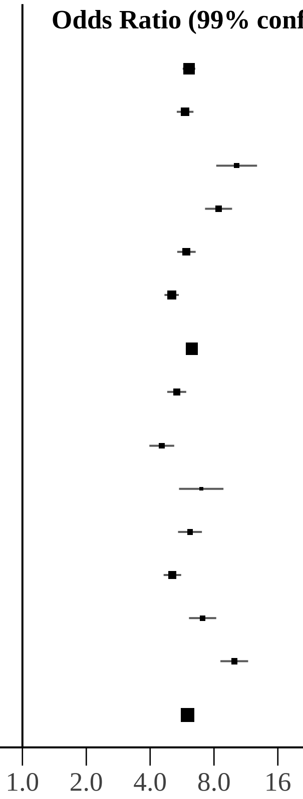

B. Diabetes and Ischemic Heart Disease

Group		Odds Ratio (99% confidence interval)	Cases	Controls
Sex	Male	1.6 (1.5, 1.8)	26,890	37,854
	Female	1.9 (1.6, 2.1)	10,915	22,595
Age (years)	30–39	1.4 (0.9, 2.3)	3,098	8,330
	40–49	1.6 (1.2, 2.0)	6,935	10,792
	50–59	1.9 (1.6, 2.2)	12,154	16,362
	60–69	1.7 (1.5, 1.9)	15,618	24,965
Residence	Rural	1.6 (1.4, 1.8)	26,397	47,489
	Urban	1.9 (1.7, 2.2)	11,408	12,959
Region	North	1.6 (1.3, 2.1)	6,588	8,641
	Northeast	1.4 (0.9, 2.4)	1,109	4,608
	East	1.2 (1.0, 1.5)	4,768	10,711
	South	1.8 (1.6, 2.1)	14,771	16,562
	West	2.6 (2.0, 3.2)	5,763	7,482
	Central	1.6 (1.2, 2.3)	4,806	12,445
Total		1.7 (1.6, 1.9)	37,805	60,449

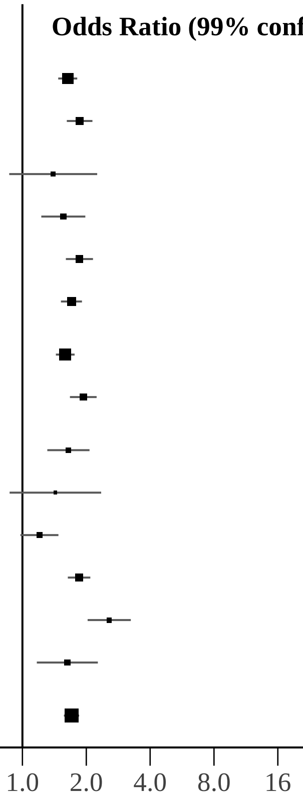

eFigure 8 Association between hypertension (A,C) or diabetes (B,D) and stroke mortality in high-burden (A,B) and low-burden (C,D) states, where both physicians initially agreed on cause of death category without further adjudication. The total and stratified estimates are adjusted for age, sex, urban/rural residence, region, smoking, alcohol use, and year*. The area of each box is proportional to the sample size (cases and controls).

*We tested whether the effects of diabetes and hypertension varied by year. There was a significant interaction between diabetes and year in low-burden states only (p=0.002), and between hypertension and year in both high- and low-burden states (p=0.002 and p=0.01 respectively). These interaction terms were included in all models.

A. Hypertension and Stroke, High-Burden States

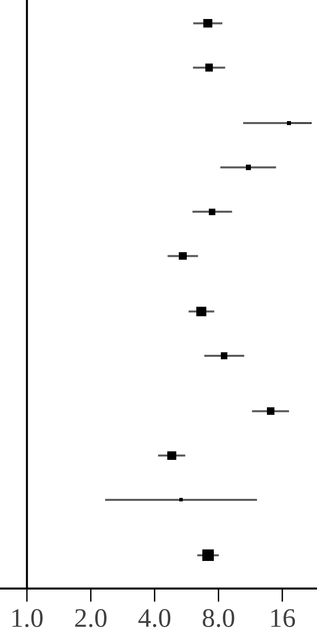

Group		Odds Ratio (99% confidence interval)	Cases	Controls
Sex	Male	7.1 (6.1, 8.3)	3,725	6,454
	Female	7.2 (6.1, 8.6)	3,095	4,767
Age (years)	30–39	17.2 (10.5, 28.3)	362	1,601
	40–49	11.0 (8.2, 15.0)	953	2,191
	50–59	7.5 (6.0, 9.3)	2,113	3,141
	60–69	5.4 (4.6, 6.4)	3,392	4,288
Residence	Rural	6.6 (5.8, 7.6)	4,649	7,980
	Urban	8.5 (6.9, 10.6)	2,171	3,241
Region	Northeast	14.1 (11.5, 17.2)	2,132	4,608
	East	4.8 (4.2, 5.6)	4,214	5,773
	Central	5.3 (2.3, 12.2)	474	840
Total		7.1 (6.4, 8.0)	6,820	11,221

B. Diabetes and Stroke, High-Burden States

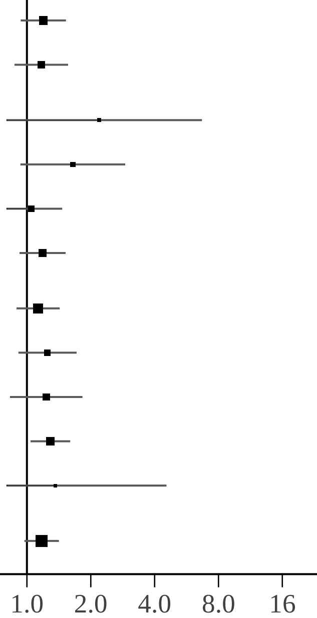

Group		Odds Ratio (99% confidence interval)	Cases	Controls
Sex	Male	1.2 (0.9, 1.5)	3,725	6,454
	Female	1.2 (0.9, 1.6)	3,095	4,767
Age (years)	30–39	2.2 (0.7, 6.7)	362	1,601
	40–49	1.6 (0.9, 2.9)	953	2,191
	50–59	1.0 (0.7, 1.5)	2,113	3,141
	60–69	1.2 (0.9, 1.5)	3,392	4,288
Residence	Rural	1.1 (0.9, 1.4)	4,649	7,980
	Urban	1.3 (0.9, 1.7)	2,171	3,241
	Northeast	1.2 (0.8, 1.8)	2,132	4,608
	East	1.3 (1.0, 1.6)	4,214	5,773
	Central	1.4 (0.4, 4.5)	474	840
Total		1.2 (1.0, 1.4)	6,820	11,221

C. Hypertension and Stroke, Low-Burden States

Group		Odds Ratio (99% confidence interval)	Cases	Controls
Sex	Male	8.0 (7.2, 8.8)	6,996	31,400
	Female	7.5 (6.7, 8.5)	4,395	17,828
Age (years)	30–39	16.3 (11.4, 23.4)	460	6,729
	40–49	12.5 (9.9, 15.8)	1,160	8,601
	50–59	8.0 (6.9, 9.2)	3,135	13,221
	60–69	6.7 (6.0, 7.4)	6,636	20,677
Residence	Rural	7.9 (7.3, 8.7)	8,884	39,509
	Urban	7.6 (6.5, 8.8)	2,507	9,718
Region	North	7.4 (6.1, 9.0)	1,398	8,641
	East	12.9 (10.1, 16.5)	1,450	4,938
	South	10.3 (9.1, 11.6)	4,561	16,562
	West	5.1 (4.2, 6.1)	2,019	7,482
	Central	4.6 (3.7, 5.6)	1,963	11,605
Total		7.8 (7.2, 8.4)	11,391	49,228

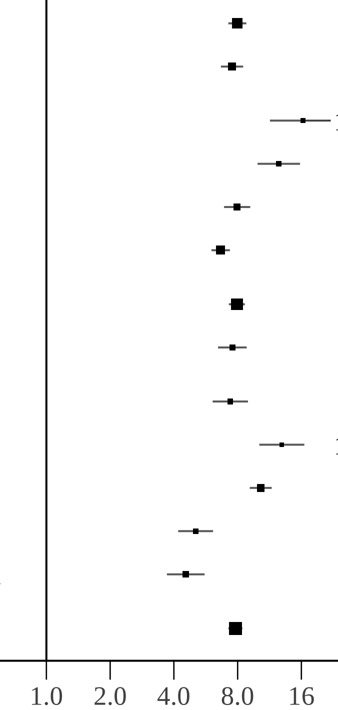

D. Diabetes and Stroke, Low-Burden States

Group		Odds Ratio (99% confidence interval)	Cases	Controls
Sex	Male	1.3 (1.1, 1.5)	6,996	31,400
	Female	1.4 (1.1, 1.7)	4,395	17,828
Age (years)	30–39	0.9 (0.4, 2.2)	460	6,729
	40–49	1.1 (0.7, 1.7)	1,160	8,601
	50–59	1.5 (1.2, 1.9)	3,135	13,221
	60–69	1.3 (1.1, 1.5)	6,636	20,677
Residence	Rural	1.3 (1.1, 1.5)	8,884	39,509
	Urban	1.5 (1.2, 1.8)	2,507	9,718
Region	North	1.1 (0.7, 1.5)	1,398	8,641
	East	1.0 (0.6, 1.6)	1,450	4,938
	South	1.2 (1.0, 1.4)	4,561	16,562
	West	1.8 (1.4, 2.5)	2,019	7,482
	Central	1.6 (1.1, 2.5)	1,963	11,605
Total		1.3 (1.2, 1.5)	11,391	49,228

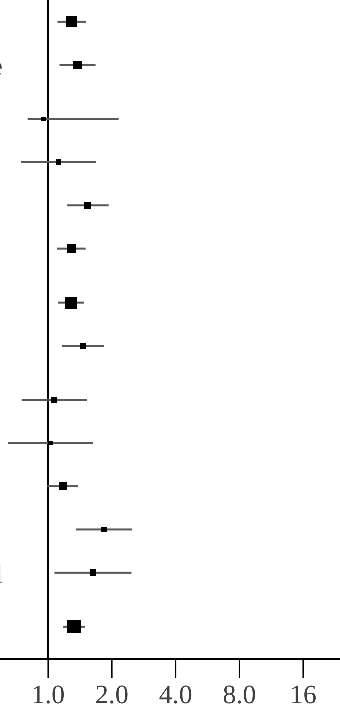

eFigure 9 Total and stratified estimates of the association between (A) hypertension or (B) diabetes and ischemic heart disease mortality, displayed separately for men and women. Estimates are adjusted for age, rurality, region, smoking, alcohol use, and year. The area of each box is proportional to the sample size (cases and controls).

A. Hypertension and Ischemic Heart Disease

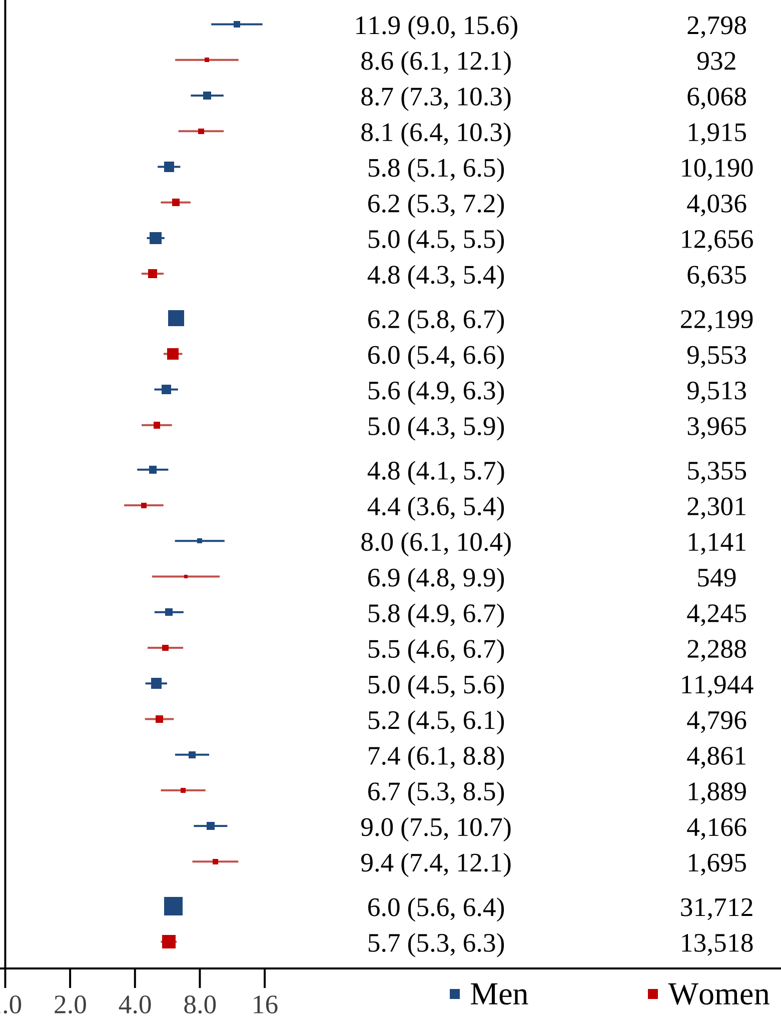

Group		Odds Ratio (99% confidence interval)	Cases	Controls
Age (years)	30–39	11.9 (9.0, 15.6)	2,798	5,769
		8.6 (6.1, 12.1)	932	2,561
	40–49	8.7 (7.3, 10.3)	6,068	6,979
		8.1 (6.4, 10.3)	1,915	3,813
	50–59	5.8 (5.1, 6.5)	10,190	10,116
		6.2 (5.3, 7.2)	4,036	6,246
	60–69	5.0 (4.5, 5.5)	12,656	14,990
		4.8 (4.3, 5.4)	6,635	9,975
Residence	Rural	6.2 (5.8, 6.7)	22,199	29,570
		6.0 (5.4, 6.6)	9,553	17,919
	Urban	5.6 (4.9, 6.3)	9,513	8,283
		5.0 (4.3, 5.9)	3,965	4,676
Region	North	4.8 (4.1, 5.7)	5,355	5,593
		4.4 (3.6, 5.4)	2,301	3,048
	Northeast	8.0 (6.1, 10.4)	1,141	2,937
		6.9 (4.8, 9.9)	549	1,671
	East	5.8 (4.9, 6.7)	4,245	6,315
		5.5 (4.6, 6.7)	2,288	4,396
	South	5.0 (4.5, 5.6)	11,944	10,414
		5.2 (4.5, 6.1)	4,796	6,148
	West	7.4 (6.1, 8.8)	4,861	4,606
		6.7 (5.3, 8.5)	1,889	2,876
	Central	9.0 (7.5, 10.7)	4,166	7,989
		9.4 (7.4, 12.1)	1,695	4,456
	Total	6.0 (5.6, 6.4)	31,712	37,854
		5.7 (5.3, 6.3)	13,518	22,595

B. Diabetes and Ischemic Heart Disease

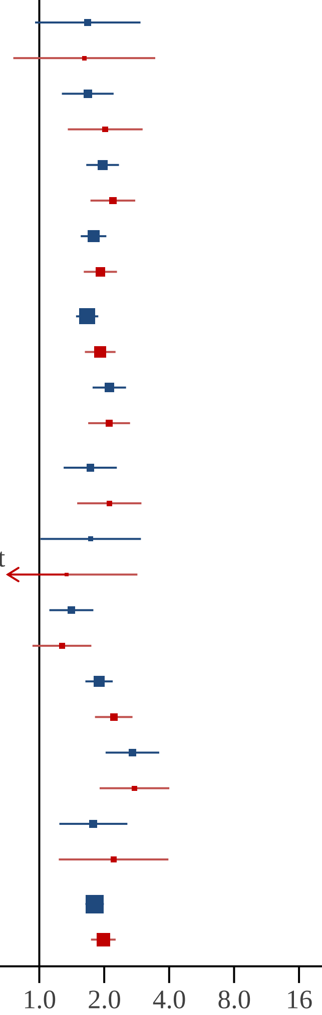

Group		Sex	Odds Ratio (99% confidence interval)	Cases	Controls
Age (years)	30–39	Men	1.7 (1.0, 2.9)	2,798	5,769
		Women	1.6 (0.8, 3.4)	932	2,561
	40–49	Men	1.7 (1.3, 2.2)	6,068	6,979
		Women	2.0 (1.4, 3.0)	1,915	3,813
	50–59	Men	2.0 (1.7, 2.3)	10,190	10,116
		Women	2.2 (1.7, 2.8)	4,036	6,246
	60–69	Men	1.8 (1.6, 2.0)	12,656	14,990
		Women	1.9 (1.6, 2.3)	6,635	9,975
Residence	Rural	Men	1.7 (1.5, 1.9)	22,199	29,570
		Women	1.9 (1.6, 2.3)	9,553	17,919
	Urban	Men	2.1 (1.8, 2.5)	9,513	8,283
		Women	2.1 (1.7, 2.6)	3,965	4,676
Region	North	Men	1.7 (1.3, 2.3)	5,355	5,593
		Women	2.1 (1.5, 3.0)	2,301	3,048
	Northeast	Men	1.7 (1.0, 3.0)	1,141	2,937
		Women	1.3 (0.6, 2.9)	549	1,671
	East	Men	1.4 (1.1, 1.8)	4,245	6,315
		Women	1.3 (0.9, 1.7)	2,288	4,396
	South	Men	1.9 (1.6, 2.2)	11,944	10,414
		Women	2.2 (1.8, 2.7)	4,796	6,148
	West	Men	2.7 (2.0, 3.6)	4,861	4,606
		Women	2.8 (1.9, 4.0)	1,889	2,876
	Central	Men	1.8 (1.2, 2.6)	4,166	7,989
		Women	2.2 (1.2, 4.0)	1,695	4,456
Total		Men	1.8 (1.6, 2.0)	31,712	37,854
		Women	2.0 (1.7, 2.3)	13,518	22,595

■ Men ■ Women

eFigure 10 Total and stratified estimates of the association between (A) hypertension or (B) diabetes and stroke mortality in high-burden states, displayed separately for men and women. Estimates are adjusted for age, rurality, region, smoking, alcohol use, and year. The area of each box is proportional to the sample size (cases and controls).

A. Hypertension and Stroke, High-Burden States

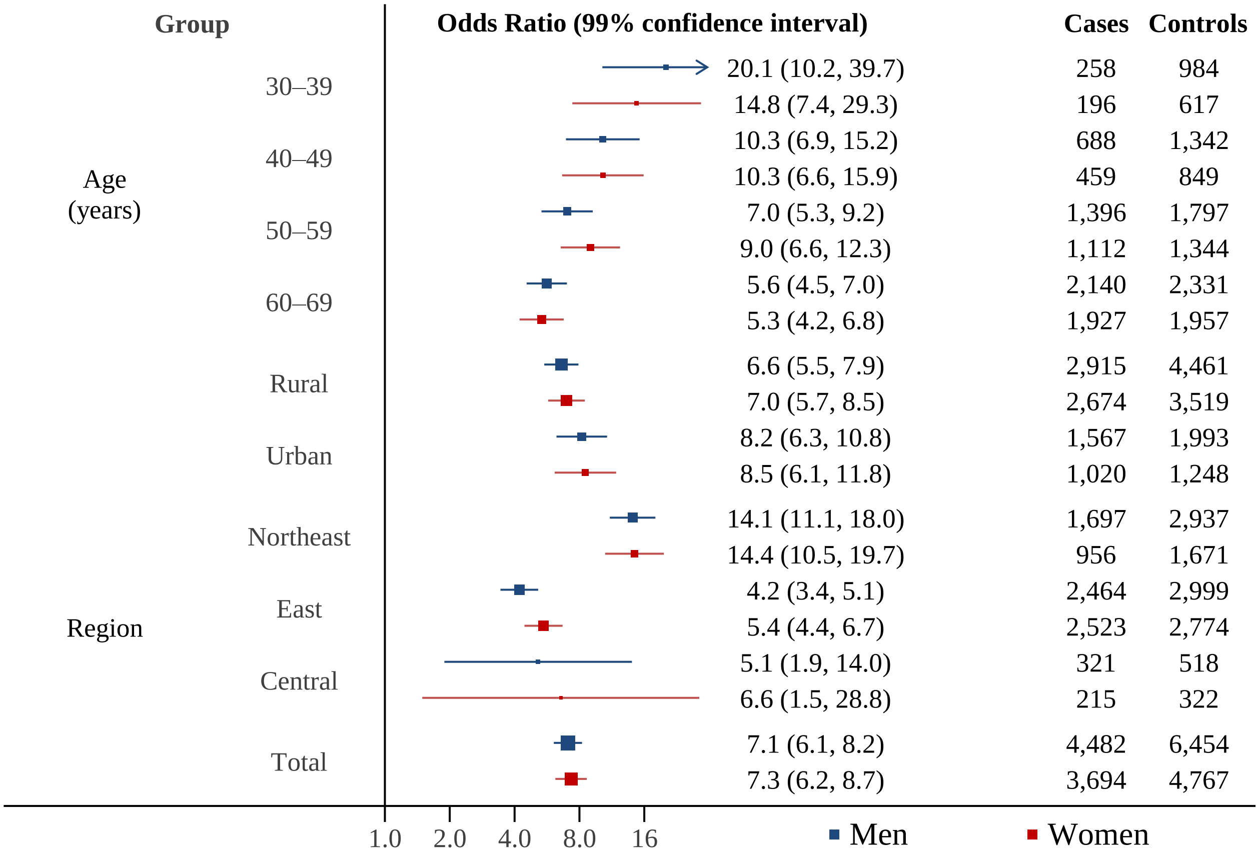

B. Diabetes and Stroke, High-Burden States

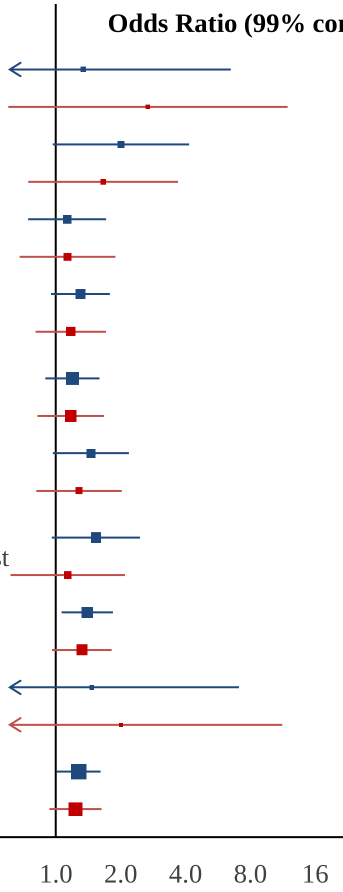

Group		Odds Ratio (99% confidence interval)	Cases	Controls
Age (years)	30–39	1.3 (0.3, 6.5)	258	984
		2.7 (0.6, 11.9)	196	617
	40–49	2.0 (1.0, 4.2)	688	1,342
		1.7 (0.7, 3.7)	459	849
	50–59	1.1 (0.7, 1.7)	1,396	1,797
		1.1 (0.7, 1.9)	1,112	1,344
	60–69	1.3 (1.0, 1.8)	2,140	2,331
		1.2 (0.8, 1.7)	1,927	1,957
	Rural	1.2 (0.9, 1.6)	2,915	4,461
		1.2 (0.8, 1.7)	2,674	3,519
	Urban	1.5 (1.0, 2.2)	1,567	1,993
		1.3 (0.8, 2.0)	1,020	1,248
Region	Northeast	1.5 (1.0, 2.5)	1,697	2,937
		1.1 (0.6, 2.1)	956	1,671
	East	1.4 (1.1, 1.8)	2,464	2,999
		1.3 (1.0, 1.8)	2,523	2,774
	Central	1.5 (0.3, 7.1)	321	518
		2.0 (0.4, 11.3)	215	322
	Total	1.3 (1.0, 1.6)	4,482	6,454
		1.2 (0.9, 1.6)	3,694	4,767

eFigure 11 Total and stratified estimates of the association between (A) hypertension or (B) diabetes and stroke mortality in low-burden states, displayed separately for men and women. Estimates are adjusted for age, rurality, region, smoking, alcohol use, and year. The area of each box is proportional to the sample size (cases and controls).

Abbreviation: N/A, cannot be estimated due to limited data

A. Hypertension and Stroke, Low-Burden

Group		Odds Ratio (99% confidence interval)	Cases	Controls
Age (years)	30–39	17.6 (11.4, 27.1)	411	4,785
		N/A	219	1,944
	40–49	12.4 (9.5, 16.2)	1,010	5,637
		12.6 (8.8, 17.9)	523	2,964
	50–59	8.2 (6.9, 9.6)	2,535	8,319
		8.0 (6.4, 9.9)	1,383	4,902
	60–69	7.0 (6.2, 7.9)	4,806	12,659
		6.4 (5.5, 7.4)	3,388	8,018
Residence	Rural	8.4 (7.6, 9.4)	6,826	25,109
		7.4 (6.5, 8.5)	4,275	14,400
	Urban	7.6 (6.3, 9.2)	1,936	6,290
		7.9 (6.2, 9.9)	1,238	3,428
Region	North	7.9 (6.2, 9.9)	1,098	5,593
		N/A	763	3,048
	East	14.1 (10.7, 18.5)	1,451	3,316
		10.7 (7.0, 16.6)	485	1,622
	South	10.6 (9.1, 12.2)	3,387	10,414
		9.6 (8.1, 11.5)	2,246	6,148
	West	5.0 (3.9, 6.3)	1,496	4,606
		5.5 (4.2, 7.3)	1,000	2,876
	Central	4.8 (3.8, 6.2)	1,330	7,471
		5.0 (3.7, 6.7)	1,019	4,134
	Total	8.2 (7.5, 9.0)	8,762	31,400
		7.5 (6.7, 8.3)	5,513	17,828

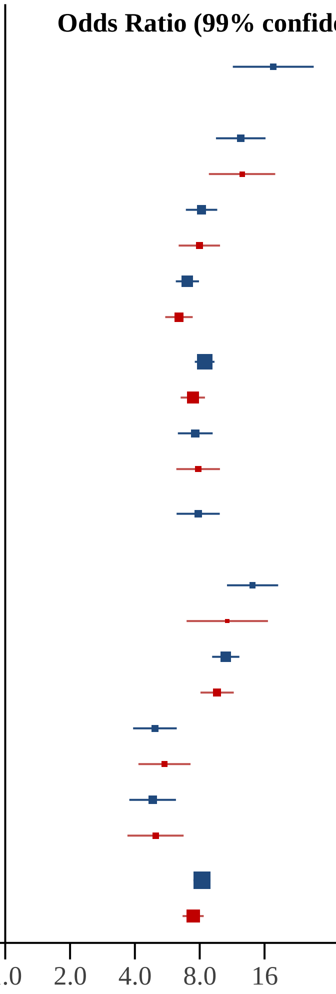

■ Men ■ Women

B. Diabetes and Stroke, Low-Burden

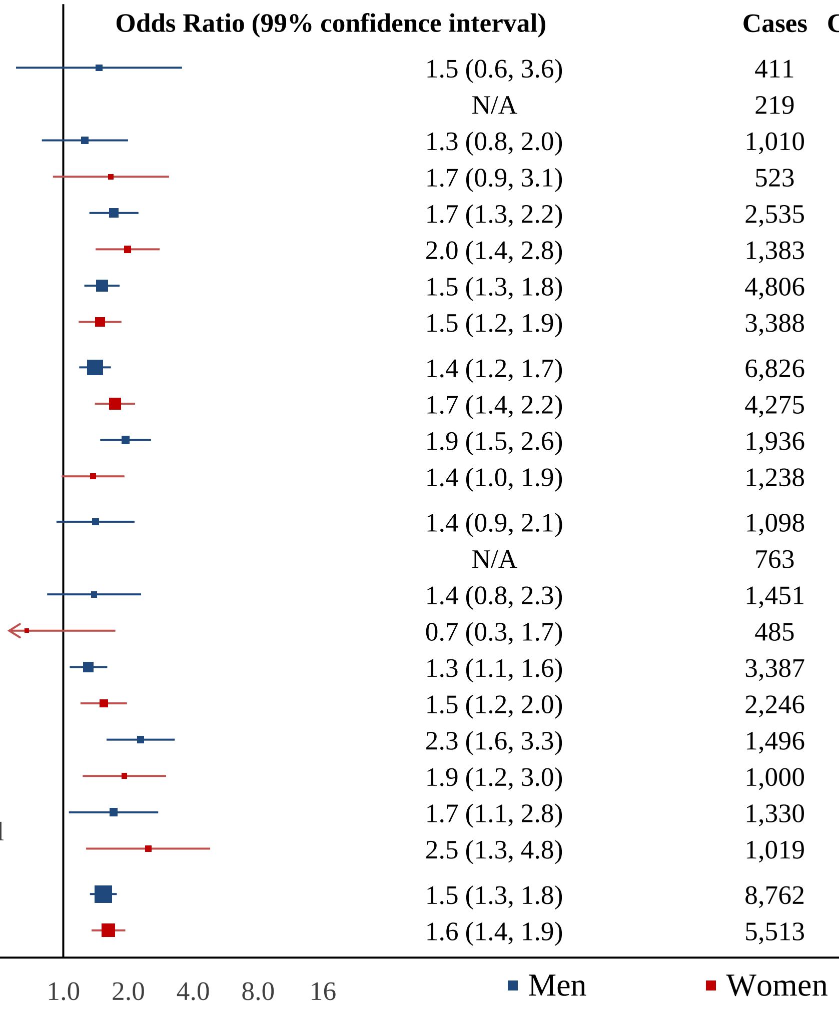

Group		Sex	Odds Ratio (99% confidence interval)	Cases	Controls
Age (years)	30–39	Men	1.5 (0.6, 3.6)	411	4,785
		Women	N/A	219	1,944
	40–49	Men	1.3 (0.8, 2.0)	1,010	5,637
		Women	1.7 (0.9, 3.1)	523	2,964
	50–59	Men	1.7 (1.3, 2.2)	2,535	8,319
		Women	2.0 (1.4, 2.8)	1,383	4,902
	60–69	Men	1.5 (1.3, 1.8)	4,806	12,659
		Women	1.5 (1.2, 1.9)	3,388	8,018
Residence	Rural	Men	1.4 (1.2, 1.7)	6,826	25,109
		Women	1.7 (1.4, 2.2)	4,275	14,400
	Urban	Men	1.9 (1.5, 2.6)	1,936	6,290
		Women	1.4 (1.0, 1.9)	1,238	3,428
Region	North	Men	1.4 (0.9, 2.1)	1,098	5,593
		Women	N/A	763	3,048
	East	Men	1.4 (0.8, 2.3)	1,451	3,316
		Women	0.7 (0.3, 1.7)	485	1,622
	South	Men	1.3 (1.1, 1.6)	3,387	10,414
		Women	1.5 (1.2, 2.0)	2,246	6,148
	West	Men	2.3 (1.6, 3.3)	1,496	4,606
		Women	1.9 (1.2, 3.0)	1,000	2,876
	Central	Men	1.7 (1.1, 2.8)	1,330	7,471
		Women	2.5 (1.3, 4.8)	1,019	4,134
Total		Men	1.5 (1.3, 1.8)	8,762	31,400
		Women	1.6 (1.4, 1.9)	5,513	17,828

eFigure 12 Total and stratified estimates of the association between (A) hypertension or (B) diabetes and ischemic heart disease mortality, with and without socioeconomic status (SES) adjustment (2001 to 2013 only). Estimates are adjusted for age, sex, rurality, region, smoking, alcohol use, and year. The area of each box is proportional to the sample size (cases and controls).

A. Hypertension and Ischemic Heart Disease

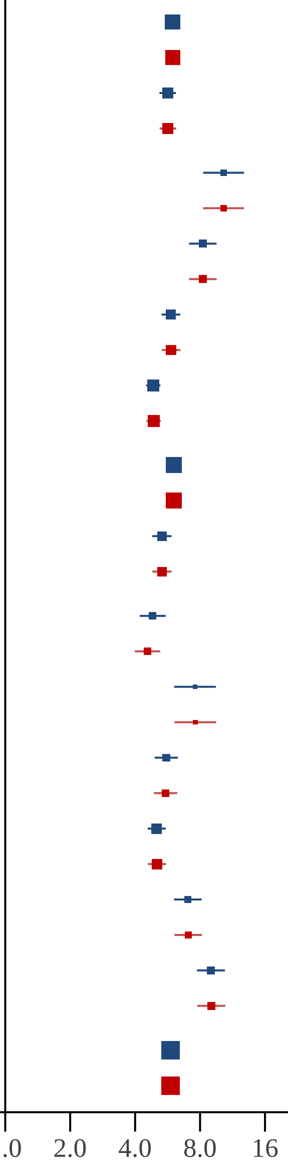

Group		Odds Ratio (99% confidence interval)	Cases	Controls
Sex	Male	6.0 (5.6, 6.4)	29,388	35,698
		6.0 (5.6, 6.4)	29,388	35,698
	Female	5.7 (5.2, 6.2)	12,601	21,464
		5.7 (5.2, 6.2)	12,601	21,464
Age (years)	30–39	10.3 (8.3, 12.8)	3,487	7,904
		10.3 (8.3, 12.8)	3,487	7,904
	40–49	8.2 (7.1, 9.5)	7,450	10,180
		8.2 (7.1, 9.6)	7,450	10,180
	50–59	5.9 (5.3, 6.5)	13,089	15,338
		5.9 (5.3, 6.5)	13,089	15,338
	60–69	4.9 (4.5, 5.2)	17,963	23,740
		4.9 (4.5, 5.3)	17,963	23,740
Residence	Rural	6.0 (5.7, 6.4)	29,294	44,885
		6.1 (5.7, 6.5)	29,294	44,885
	Urban	5.3 (4.8, 5.9)	12,695	12,276
		5.3 (4.8, 5.9)	12,695	12,276
Region	North	4.8 (4.2, 5.5)	6,958	7,993
		4.6 (4.0, 5.2)	6,958	7,993
	Northeast	7.6 (6.1, 9.5)	1,531	4,325
		7.6 (6.1, 9.5)	1,531	4,325
	East	5.6 (4.9, 6.3)	6,078	10,157
		5.5 (4.9, 6.3)	6,078	10,157
	South	5.0 (4.6, 5.5)	15,644	15,642
		5.0 (4.6, 5.6)	15,644	15,642
	West	7.0 (6.1, 8.1)	6,297	7,150
		7.0 (6.1, 8.2)	6,297	7,150
	Central	9.0 (7.7, 10.4)	5,481	11,895
		9.0 (7.8, 10.5)	5,481	11,895
	Total	5.8 (5.5, 6.2)	41,989	57,162
		5.9 (5.5, 6.2)	41,989	57,162

B. Diabetes and Ischemic Heart Disease

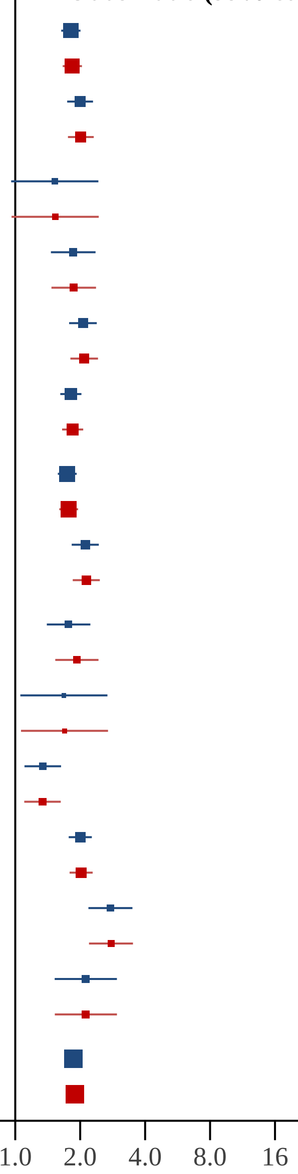

Group			Odds Ratio (99% confidence interval)	Cases	Controls
Sex	Male	With SES	1.8 (1.6, 2.0)	29,388	35,698
		Without SES	1.8 (1.7, 2.0)	29,388	35,698
	Female	With SES	2.0 (1.7, 2.3)	12,601	21,464
		Without SES	2.0 (1.8, 2.3)	12,601	21,464
Age (years)	30–39	With SES	1.5 (1.0, 2.4)	3,487	7,904
		Without SES	1.5 (1.0, 2.4)	3,487	7,904
	40–49	With SES	1.9 (1.5, 2.4)	7,450	10,180
		Without SES	1.9 (1.5, 2.4)	7,450	10,180
	50–59	With SES	2.1 (1.8, 2.4)	13,089	15,338
		Without SES	2.1 (1.8, 2.4)	13,089	15,338
	60–69	With SES	1.8 (1.6, 2.0)	17,963	23,740
		Without SES	1.8 (1.6, 2.1)	17,963	23,740
Residence	Rural	With SES	1.7 (1.6, 1.9)	29,294	44,885
		Without SES	1.8 (1.6, 2.0)	29,294	44,885
	Urban	With SES	2.1 (1.8, 2.4)	12,695	12,276
		Without SES	2.1 (1.8, 2.5)	12,695	12,276
Region	North	With SES	1.8 (1.4, 2.2)	6,958	7,993
		Without SES	1.9 (1.5, 2.4)	6,958	7,993
	Northeast	With SES	1.7 (1.1, 2.7)	1,531	4,325
		Without SES	1.7 (1.1, 2.7)	1,531	4,325
	East	With SES	1.3 (1.1, 1.6)	6,078	10,157
		Without SES	1.3 (1.1, 1.6)	6,078	10,157
	South	With SES	2.0 (1.8, 2.3)	15,644	15,642
		Without SES	2.0 (1.8, 2.3)	15,644	15,642
	West	With SES	2.8 (2.2, 3.5)	6,297	7,150
		Without SES	2.8 (2.2, 3.5)	6,297	7,150
	Central	With SES	2.1 (1.5, 3.0)	5,481	11,895
		Without SES	2.1 (1.5, 3.0)	5,481	11,895
	Total	With SES	1.9 (1.7, 2.0)	41,989	57,162
		Without SES	1.9 (1.7, 2.0)	41,989	57,162

eFigure 13 Total and stratified estimates of the association between (A) hypertension or (B) diabetes and stroke mortality in high-burden states, with and without socioeconomic status (SES) adjustment (2001 to 2013 only). Estimates are adjusted for age, sex, rurality, region, smoking, alcohol use, and year. The area of each box is proportional to the sample size (cases and controls).

A. Hypertension and Stroke, High-Burden

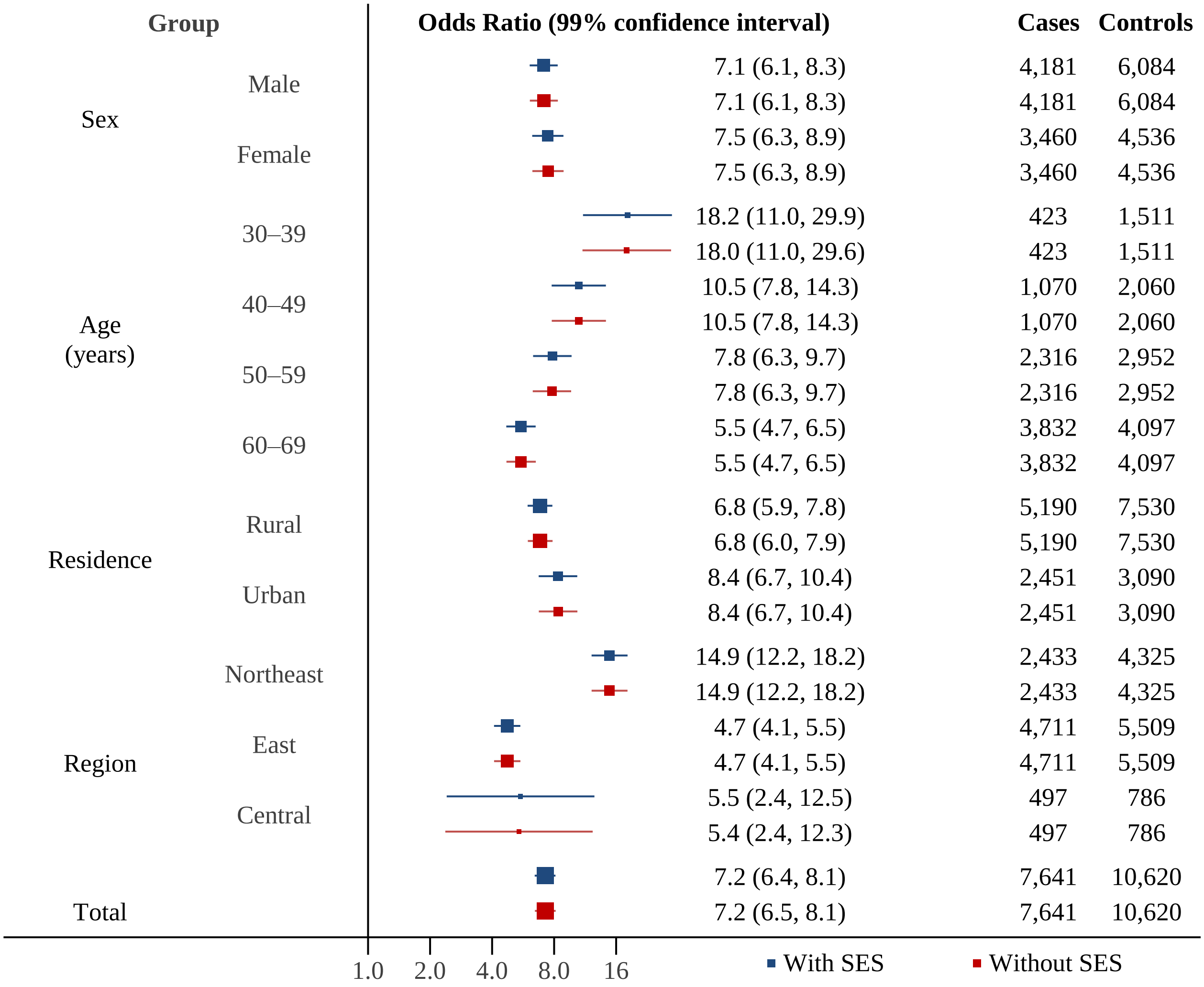

B. Diabetes and Stroke, High-Burden

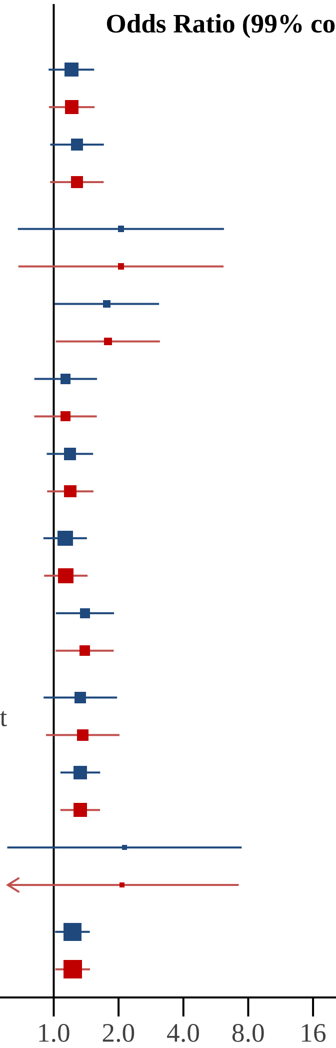

Group		Odds Ratio (99% confidence interval)	Cases	Controls
Sex	Male	1.2 (0.9, 1.5)	4,181	6,084
		1.2 (1.0, 1.5)	4,181	6,084
	Female	1.3 (1.0, 1.7)	3,460	4,536
		1.3 (1.0, 1.7)	3,460	4,536
Age (years)	30–39	2.1 (0.7, 6.2)	423	1,511
		2.1 (0.7, 6.1)	423	1,511
	40–49	1.8 (1.0, 3.1)	1,070	2,060
		1.8 (1.0, 3.1)	1,070	2,060
	50–59	1.1 (0.8, 1.6)	2,316	2,952
		1.1 (0.8, 1.6)	2,316	2,952
	60–69	1.2 (0.9, 1.5)	3,832	4,097
		1.2 (0.9, 1.5)	3,832	4,097
Residence	Rural	1.1 (0.9, 1.4)	5,190	7,530
		1.1 (0.9, 1.4)	5,190	7,530
	Urban	1.4 (1.0, 1.9)	2,451	3,090
		1.4 (1.0, 1.9)	2,451	3,090
Region	Northeast	1.3 (0.9, 2.0)	2,433	4,325
		1.4 (0.9, 2.0)	2,433	4,325
	East	1.3 (1.1, 1.6)	4,711	5,509
		1.3 (1.1, 1.6)	4,711	5,509
	Central	2.1 (0.6, 7.5)	497	786
		2.1 (0.6, 7.2)	497	786
Total		1.2 (1.0, 1.5)	7,641	10,620
		1.2 (1.0, 1.5)	7,641	10,620

eFigure 14 Total and stratified estimates of the association between (A) hypertension or (B) diabetes and stroke mortality in low-burden states, with and without socioeconomic status (SES) adjustment (2001 to 2013 only). Estimates are adjusted for age, sex, rurality, region, smoking, alcohol use, and year. The area of each box is proportional to the sample size (cases and controls).

A. Hypertension and Stroke, Low-Burden

Group		Odds Ratio (99% confidence interval)	Cases	Controls
Sex	Male	8.3 (7.6, 9.1)	8,309	29,614
		8.3 (7.5, 9.1)	8,309	29,614
	Female	7.6 (6.7, 8.5)	5,259	16,928
		7.5 (6.7, 8.5)	5,259	16,928
Age (years)	30–39	14.8 (10.5, 20.7)	598	6,393
		14.7 (10.5, 20.6)	598	6,393
	40–49	12.5 (10.1, 15.6)	1,467	8,120
		12.5 (10.0, 15.5)	1,467	8,120
	50–59	8.2 (7.1, 9.4)	3,696	12,386
		8.1 (7.1, 9.3)	3,696	12,386
	60–69	6.9 (6.3, 7.6)	7,807	19,643
		6.9 (6.2, 7.6)	7,807	19,643
Residence	Rural	8.1 (7.5, 8.9)	10,540	37,355
		8.1 (7.4, 8.8)	10,540	37,355
	Urban	7.9 (6.8, 9.1)	3,028	9,186
		7.9 (6.8, 9.1)	3,028	9,186
Region	North	7.7 (6.4, 9.3)	1,751	7,993
		7.6 (6.3, 9.1)	1,751	7,993
	East	13.7 (10.8, 17.5)	1,843	4,648
		13.6 (10.7, 17.4)	1,843	4,648
	South	10.4 (9.3, 11.7)	5,392	15,642
		10.4 (9.2, 11.6)	5,392	15,642
	West	5.3 (4.4, 6.3)	2,363	7,150
		5.3 (4.4, 6.3)	2,363	7,150
	Central	4.9 (4.0, 6.0)	2,219	11,109
		4.9 (4.0, 6.0)	2,219	11,109
Total		8.0 (7.5, 8.6)	13,568	46,542
		8.0 (7.4, 8.6)	13,568	46,542

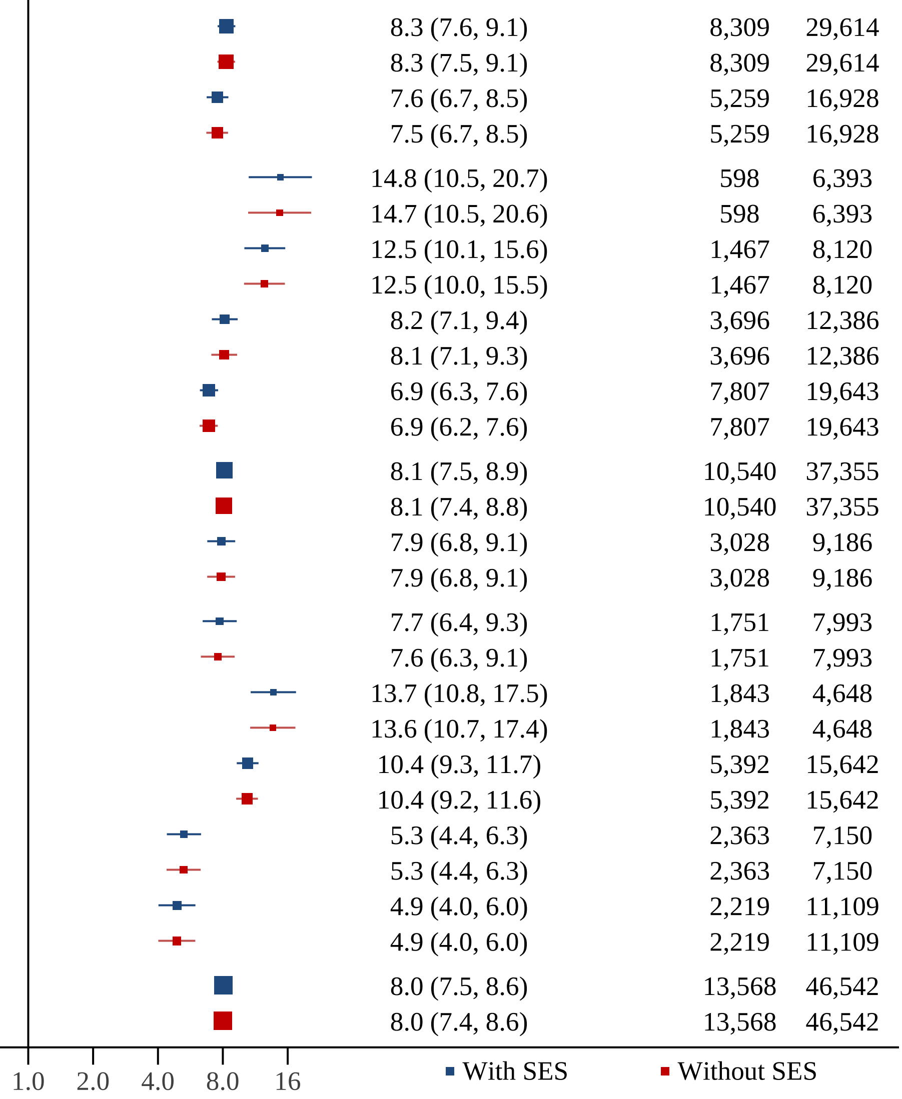

B. Diabetes and Stroke, Low-Burden

Group		Odds Ratio (99% confidence interval)	Cases	Controls
Sex	Male	1.6 (1.3, 1.8)	8,309	29,614
		1.6 (1.3, 1.8)	8,309	29,614
	Female	1.7 (1.4, 2.0)	5,259	16,928
		1.7 (1.4, 2.0)	5,259	16,928
Age (years)	30–39	1.3 (0.6, 2.6)	598	6,393
		1.3 (0.6, 2.7)	598	6,393
	40–49	1.4 (1.0, 2.1)	1,467	8,120
		1.4 (1.0, 2.1)	1,467	8,120
	50–59	1.9 (1.5, 2.3)	3,696	12,386
		1.8 (1.5, 2.3)	3,696	12,386
	60–69	1.5 (1.3, 1.8)	7,807	19,643
		1.5 (1.3, 1.8)	7,807	19,643
Residence	Rural	1.6 (1.4, 1.8)	10,540	37,355
		1.6 (1.4, 1.8)	10,540	37,355
	Urban	1.7 (1.3, 2.1)	3,028	9,186
		1.7 (1.4, 2.1)	3,028	9,186
Region	North	1.4 (1.0, 1.9)	1,751	7,993
		1.4 (1.0, 2.0)	1,751	7,993
	East	1.3 (0.8, 2.1)	1,843	4,648
		1.3 (0.8, 2.1)	1,843	4,648
	South	1.4 (1.2, 1.7)	5,392	15,642
		1.4 (1.2, 1.7)	5,392	15,642
	West	2.1 (1.6, 2.8)	2,363	7,150
		2.1 (1.5, 2.8)	2,363	7,150
	Central	2.2 (1.5, 3.3)	2,219	11,109
		2.2 (1.4, 3.3)	2,219	11,109
Total		1.6 (1.4, 1.8)	13,568	46,542
		1.6 (1.4, 1.8)	13,568	46,542

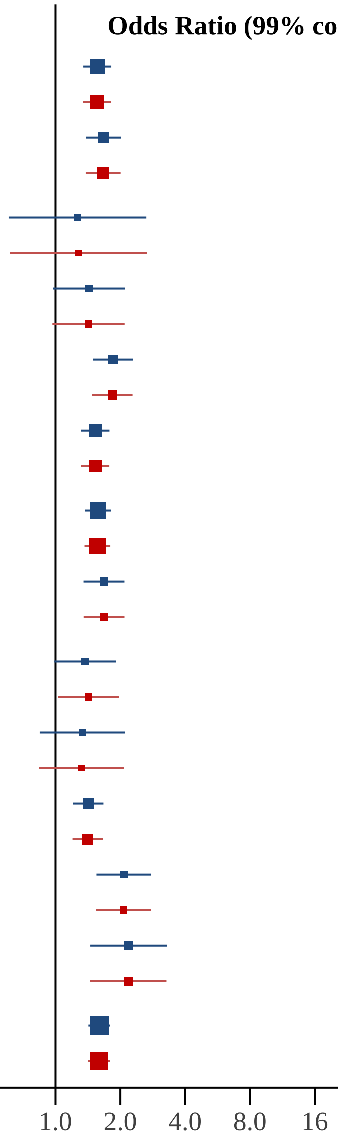

▪ With SES ▪ Without SES

eFigure 15 Total and stratified estimates of the association between (A) hypertension or (B) diabetes and ischemic heart disease mortality, with and without adjustment for clustering at the district level (2001 to 2013 only). Estimates are adjusted for age, sex, rurality, region, smoking, alcohol use, and year. The area of each box is proportional to the sample size (cases and controls).

A. Hypertension and Ischemic Heart Disease

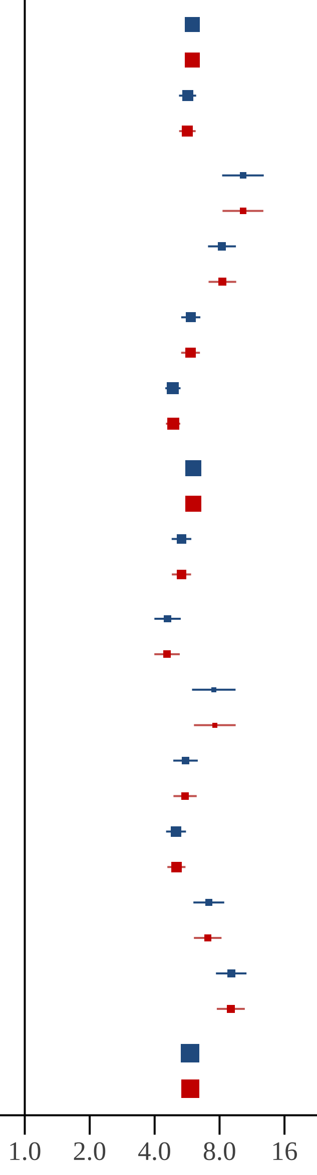

Group		Odds Ratio (99% confidence interval)	Cases	Controls
Sex	Male	6.0 (5.6, 6.4)	29,388	35,698
		6.0 (5.6, 6.4)	29,388	35,698
	Female	5.7 (5.2, 6.2)	12,601	21,464
		5.7 (5.2, 6.2)	12,601	21,464
Age (years)	30–39	10.3 (8.2, 12.8)	3,487	7,904
		10.3 (8.3, 12.8)	3,487	7,904
	40–49	8.2 (7.1, 9.5)	7,450	10,180
		8.2 (7.1, 9.6)	7,450	10,180
	50–59	5.9 (5.3, 6.5)	13,089	15,338
		5.9 (5.3, 6.5)	13,089	15,338
	60–69	4.9 (4.5, 5.3)	17,963	23,740
		4.9 (4.5, 5.3)	17,963	23,740
Residence	Rural	6.1 (5.6, 6.5)	29,294	44,885
		6.1 (5.7, 6.5)	29,294	44,885
	Urban	5.3 (4.8, 5.9)	12,695	12,276
		5.3 (4.8, 5.9)	12,695	12,276
Region	North	4.6 (4.0, 5.3)	6,958	7,993
		4.6 (4.0, 5.2)	6,958	7,993
	Northeast	7.5 (6.0, 9.5)	1,531	4,325
		7.6 (6.1, 9.5)	1,531	4,325
	East	5.6 (4.9, 6.3)	6,078	10,157
		5.5 (4.9, 6.3)	6,078	10,157
	South	5.0 (4.5, 5.6)	15,644	15,642
		5.0 (4.6, 5.6)	15,644	15,642
	West	7.1 (6.0, 8.4)	6,297	7,150
		7.0 (6.1, 8.2)	6,297	7,150
	Central	9.1 (7.7, 10.7)	5,481	11,895
		9.0 (7.8, 10.5)	5,481	11,895
	Total	5.8 (5.5, 6.2)	41,989	57,162
		5.9 (5.5, 6.2)	41,989	57,162

B. Diabetes and Ischemic Heart Disease

Group		Odds Ratio (99% confidence interval)	Cases	Controls
Sex	Male	1.8 (1.6, 2.1)	29,388	35,698
		1.8 (1.7, 2.0)	29,388	35,698
	Female	2.0 (1.7, 2.3)	12,601	21,464
		2.0 (1.8, 2.3)	12,601	21,464
Age (years)	30–39	1.5 (0.9, 2.6)	3,487	7,904
		1.5 (1.0, 2.4)	3,487	7,904
	40–49	1.9 (1.4, 2.4)	7,450	10,180
		1.9 (1.5, 2.4)	7,450	10,180
	50–59	2.1 (1.8, 2.4)	13,089	15,338
		2.1 (1.8, 2.4)	13,089	15,338
	60–69	1.8 (1.6, 2.1)	17,963	23,740
		1.8 (1.6, 2.1)	17,963	23,740
Residence	Rural	1.8 (1.6, 2.0)	29,294	44,885
		1.8 (1.6, 2.0)	29,294	44,885
	Urban	2.1 (1.8, 2.5)	12,695	12,276
		2.1 (1.8, 2.5)	12,695	12,276
Region	North	1.9 (1.4, 2.4)	6,958	7,993
		1.9 (1.5, 2.4)	6,958	7,993
	Northeast	1.7 (1.0, 2.8)	1,531	4,325
		1.7 (1.1, 2.7)	1,531	4,325
	East	1.3 (1.1, 1.7)	6,078	10,157
		1.3 (1.1, 1.6)	6,078	10,157
	South	2.1 (1.8, 2.3)	15,644	15,642
		2.0 (1.8, 2.3)	15,644	15,642
	West	2.7 (2.1, 3.4)	6,297	7,150
		2.8 (2.2, 3.5)	6,297	7,150
	Central	2.1 (1.4, 3.0)	5,481	11,895
		2.1 (1.5, 3.0)	5,481	11,895
	Total	1.9 (1.7, 2.1)	41,989	57,162
		1.9 (1.7, 2.0)	41,989	57,162

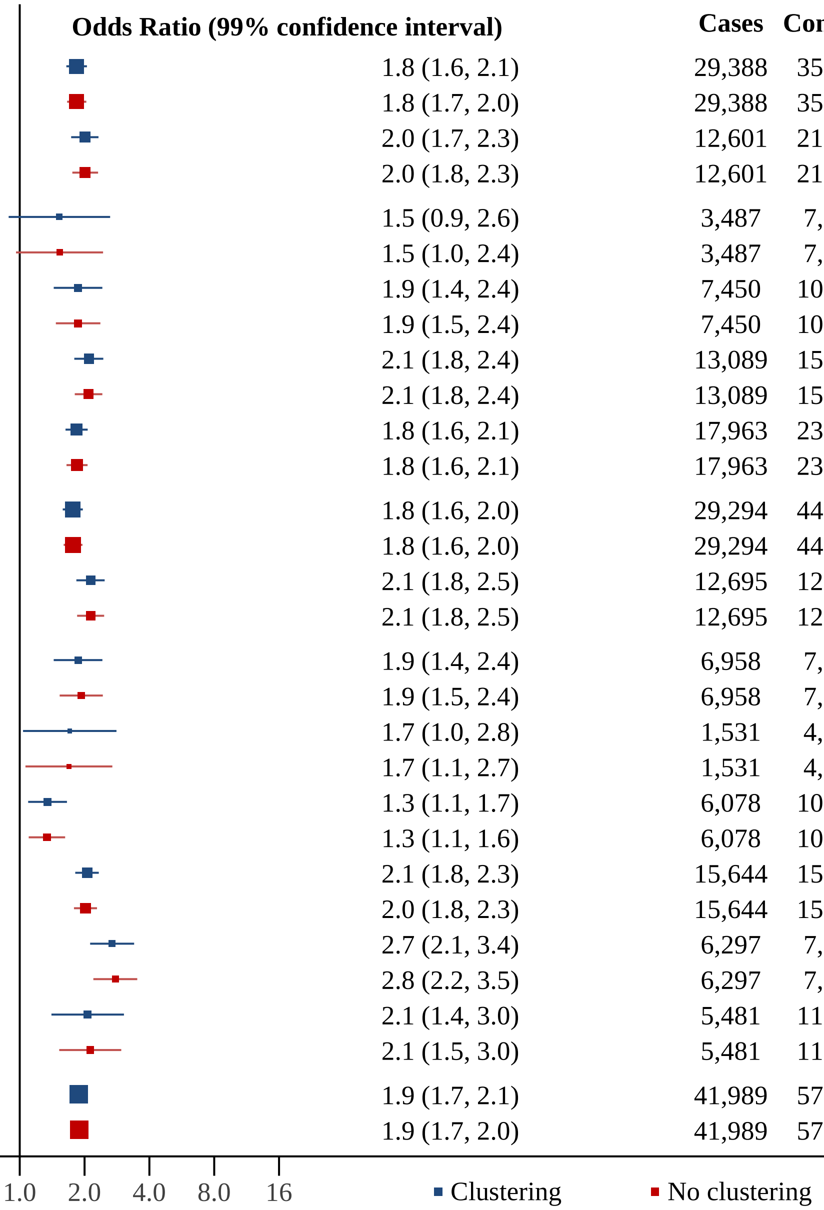

eFigure 16 Total and stratified estimates of the association between (A) hypertension or (B) diabetes and stroke mortality in high-burden states, with and without adjustment for clustering at the district level (2001 to 2013 only). Estimates are adjusted for age, sex, rurality, region, smoking, alcohol use, and year. The area of each box is proportional to the sample size (cases and controls).

A. Hypertension and Stroke, High-Burden

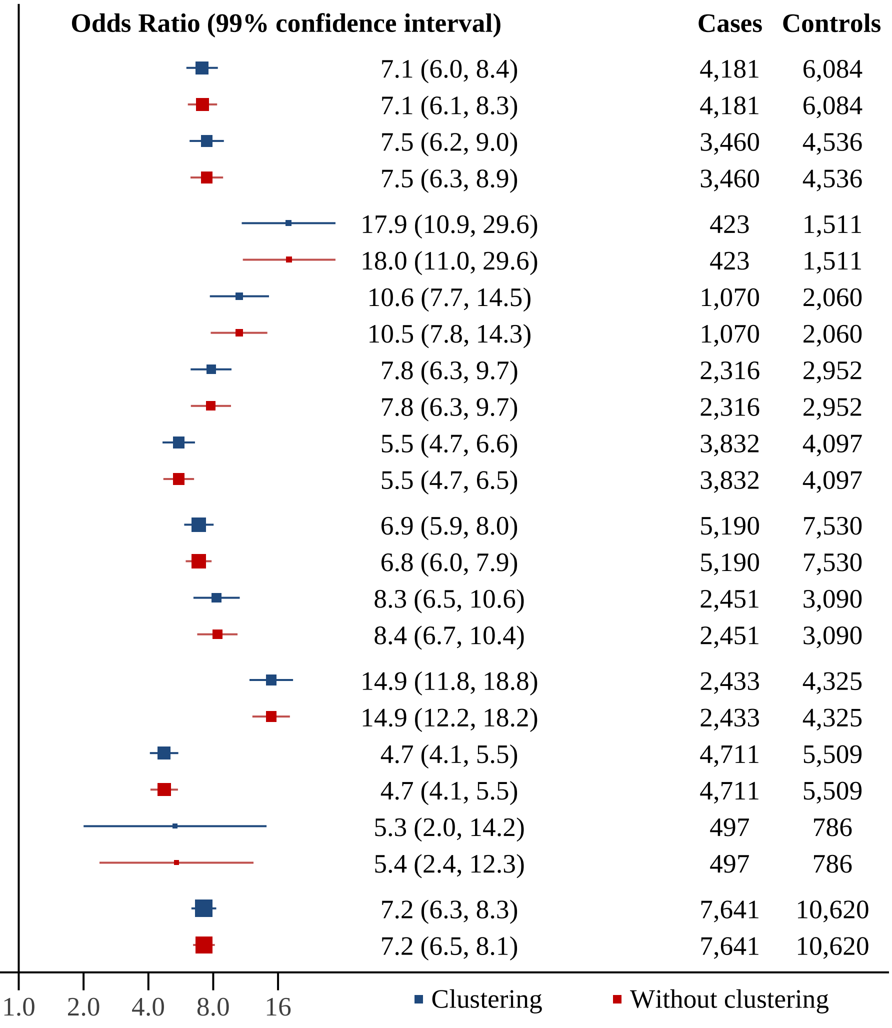

Group		Odds Ratio (99% confidence interval)	Cases	Controls
Sex	Male	7.1 (6.0, 8.4)	4,181	6,084
		7.1 (6.1, 8.3)	4,181	6,084
	Female	7.5 (6.2, 9.0)	3,460	4,536
		7.5 (6.3, 8.9)	3,460	4,536
Age (years)	30–39	17.9 (10.9, 29.6)	423	1,511
		18.0 (11.0, 29.6)	423	1,511
	40–49	10.6 (7.7, 14.5)	1,070	2,060
		10.5 (7.8, 14.3)	1,070	2,060
	50–59	7.8 (6.3, 9.7)	2,316	2,952
		7.8 (6.3, 9.7)	2,316	2,952
	60–69	5.5 (4.7, 6.6)	3,832	4,097
		5.5 (4.7, 6.5)	3,832	4,097
Residence	Rural	6.9 (5.9, 8.0)	5,190	7,530
		6.8 (6.0, 7.9)	5,190	7,530
	Urban	8.3 (6.5, 10.6)	2,451	3,090
		8.4 (6.7, 10.4)	2,451	3,090
Region	Northeast	14.9 (11.8, 18.8)	2,433	4,325
		14.9 (12.2, 18.2)	2,433	4,325
	East	4.7 (4.1, 5.5)	4,711	5,509
		4.7 (4.1, 5.5)	4,711	5,509
	Central	5.3 (2.0, 14.2)	497	786
		5.4 (2.4, 12.3)	497	786
Total		7.2 (6.3, 8.3)	7,641	10,620
		7.2 (6.5, 8.1)	7,641	10,620

B. Diabetes and Stroke, High-Burden

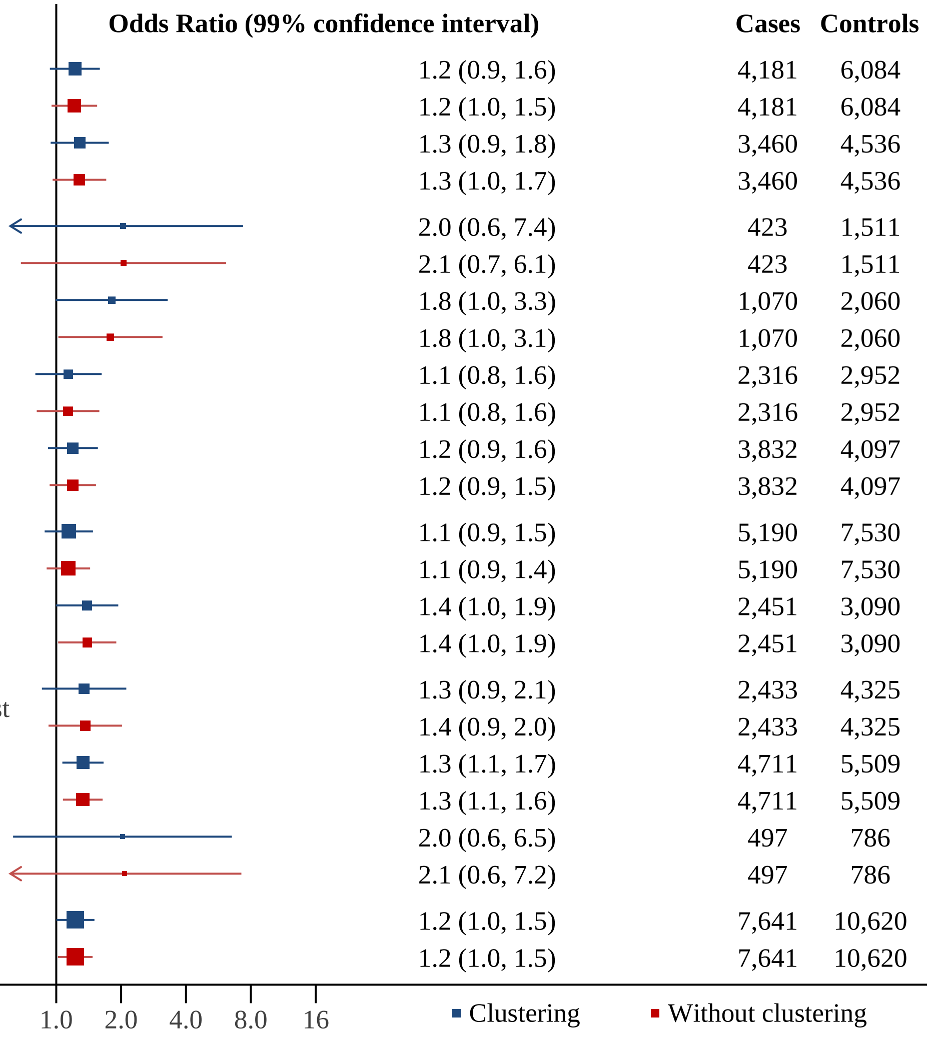

Group		Odds Ratio (99% confidence interval)	Cases	Controls
Sex	Male	1.2 (0.9, 1.6)	4,181	6,084
		1.2 (1.0, 1.5)	4,181	6,084
	Female	1.3 (0.9, 1.8)	3,460	4,536
		1.3 (1.0, 1.7)	3,460	4,536
Age (years)	30–39	2.0 (0.6, 7.4)	423	1,511
		2.1 (0.7, 6.1)	423	1,511
	40–49	1.8 (1.0, 3.3)	1,070	2,060
		1.8 (1.0, 3.1)	1,070	2,060
	50–59	1.1 (0.8, 1.6)	2,316	2,952
		1.1 (0.8, 1.6)	2,316	2,952
	60–69	1.2 (0.9, 1.6)	3,832	4,097
		1.2 (0.9, 1.5)	3,832	4,097
Residence	Rural	1.1 (0.9, 1.5)	5,190	7,530
		1.1 (0.9, 1.4)	5,190	7,530
	Urban	1.4 (1.0, 1.9)	2,451	3,090
		1.4 (1.0, 1.9)	2,451	3,090
Region	Northeast	1.3 (0.9, 2.1)	2,433	4,325
		1.4 (0.9, 2.0)	2,433	4,325
	East	1.3 (1.1, 1.7)	4,711	5,509
		1.3 (1.1, 1.6)	4,711	5,509
	Central	2.0 (0.6, 6.5)	497	786
		2.1 (0.6, 7.2)	497	786
Total		1.2 (1.0, 1.5)	7,641	10,620
		1.2 (1.0, 1.5)	7,641	10,620

eFigure 17 Total and stratified estimates of the association between (A) hypertension or (B) diabetes and stroke mortality in low-burden states, with and without adjustment for clustering at the district level (2001 to 2013 only). Estimates are adjusted for age, sex, rurality, region, smoking, alcohol use, and year. The area of each box is proportional to the sample size (cases and controls).

A. Hypertension and Stroke, Low-Burden

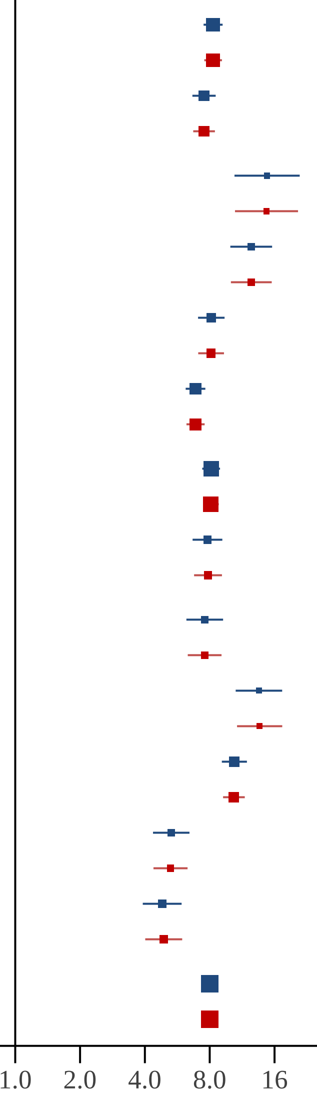

Group		Odds Ratio (99% confidence interval)	Cases	Controls
Sex	Male	8.3 (7.5, 9.2)	8,309	29,614
		8.3 (7.5, 9.1)	8,309	29,614
	Female	7.5 (6.6, 8.5)	5,259	16,928
		7.5 (6.7, 8.5)	5,259	16,928
Age (years)	30–39	14.8 (10.4, 20.9)	598	6,393
		14.7 (10.5, 20.6)	598	6,393
	40–49	12.5 (10.0, 15.6)	1,467	8,120
		12.5 (10.0, 15.5)	1,467	8,120
	50–59	8.1 (7.1, 9.4)	3,696	12,386
		8.1 (7.1, 9.3)	3,696	12,386
	60–69	6.9 (6.2, 7.6)	7,807	19,643
		6.9 (6.2, 7.6)	7,807	19,643
Residence	Rural	8.1 (7.4, 8.9)	10,540	37,355
		8.1 (7.4, 8.8)	10,540	37,355
	Urban	7.8 (6.7, 9.2)	3,028	9,186
		7.9 (6.8, 9.1)	3,028	9,186
Region	North	7.6 (6.2, 9.2)	1,751	7,993
		7.6 (6.3, 9.1)	1,751	7,993
	East	13.5 (10.6, 17.4)	1,843	4,648
		13.6 (10.7, 17.4)	1,843	4,648
	South	10.4 (9.1, 11.9)	5,392	15,642
		10.4 (9.2, 11.6)	5,392	15,642
	West	5.3 (4.4, 6.4)	2,363	7,150
		5.3 (4.4, 6.3)	2,363	7,150
	Central	4.8 (3.9, 5.9)	2,219	11,109
		4.9 (4.0, 6.0)	2,219	11,109
Total		8.0 (7.4, 8.7)	13,568	46,542
		8.0 (7.4, 8.6)	13,568	46,542

B. Diabetes and Stroke, Low-Burden

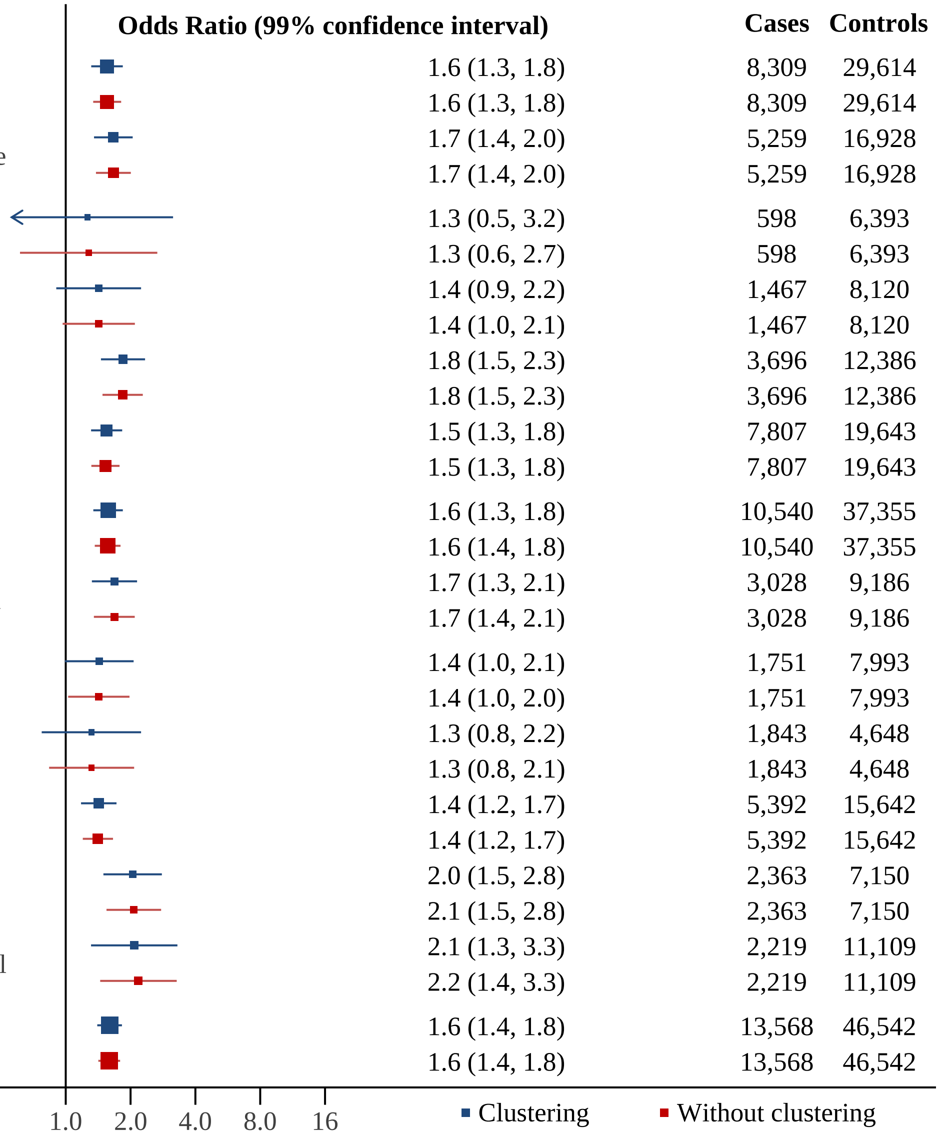

Group		Odds Ratio (99% confidence interval)	Cases	Controls
Sex	Male	1.6 (1.3, 1.8)	8,309	29,614
		1.6 (1.3, 1.8)	8,309	29,614
	Female	1.7 (1.4, 2.0)	5,259	16,928
		1.7 (1.4, 2.0)	5,259	16,928
Age (years)	30–39	1.3 (0.5, 3.2)	598	6,393
		1.3 (0.6, 2.7)	598	6,393
	40–49	1.4 (0.9, 2.2)	1,467	8,120
		1.4 (1.0, 2.1)	1,467	8,120
	50–59	1.8 (1.5, 2.3)	3,696	12,386
		1.8 (1.5, 2.3)	3,696	12,386
	60–69	1.5 (1.3, 1.8)	7,807	19,643
		1.5 (1.3, 1.8)	7,807	19,643
Residence	Rural	1.6 (1.3, 1.8)	10,540	37,355
		1.6 (1.4, 1.8)	10,540	37,355
	Urban	1.7 (1.3, 2.1)	3,028	9,186
		1.7 (1.4, 2.1)	3,028	9,186
Region	North	1.4 (1.0, 2.1)	1,751	7,993
		1.4 (1.0, 2.0)	1,751	7,993
	East	1.3 (0.8, 2.2)	1,843	4,648
		1.3 (0.8, 2.1)	1,843	4,648
	South	1.4 (1.2, 1.7)	5,392	15,642
		1.4 (1.2, 1.7)	5,392	15,642
	West	2.0 (1.5, 2.8)	2,363	7,150
		2.1 (1.5, 2.8)	2,363	7,150
	Central	2.1 (1.3, 3.3)	2,219	11,109
		2.2 (1.4, 3.3)	2,219	11,109
Total		1.6 (1.4, 1.8)	13,568	46,542
		1.6 (1.4, 1.8)	13,568	46,542

eFigure 18 Additional analysis using logistic regression to estimate the association between respondent-reported any medication usage (medication names unavailable) and mortality from ischemic heart disease and stroke among people with hypertension alone, diabetes alone, or both. Data are for 2004 onward (no available medication data for 2001–03). Estimates are adjusted for age, sex, urban or rural residence, region, smoking, alcohol use, and year. The area of each box is proportional to the sample size (cases and controls).

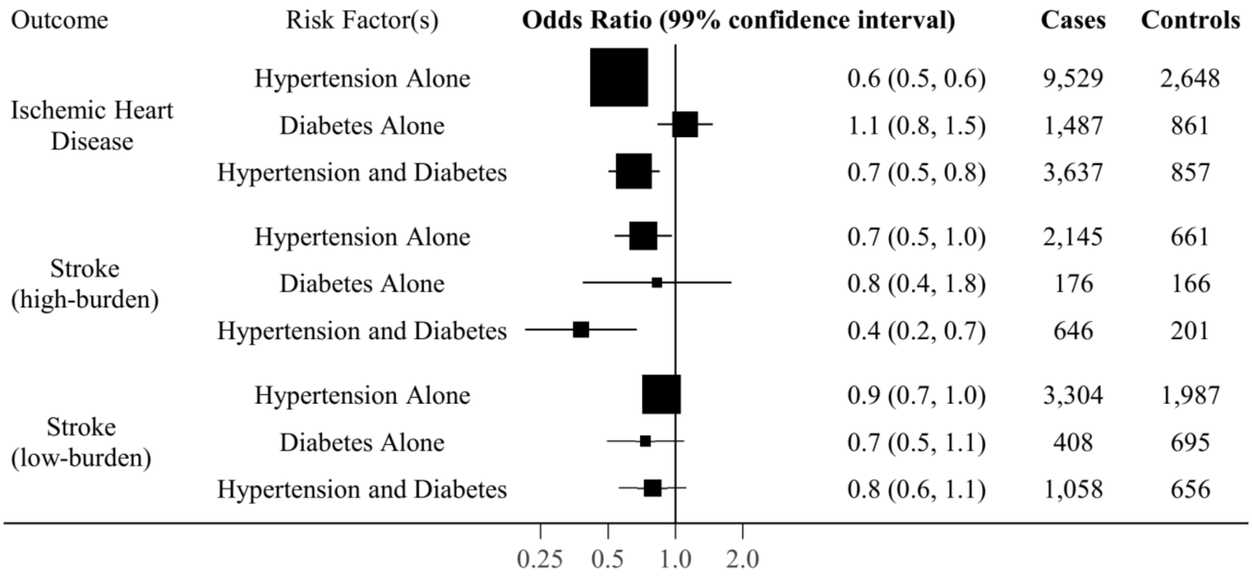

eTable 4 Sensitivity and additional analyses of the association between respondent-reported medication use and ischemic heart disease or stroke mortality among people with hypertension alone, diabetes alone, or both hypertension and diabetes. Results pertain to India as a whole; findings by state could not be generated due to sample size limitations.

		Odds Ratios (99% confidence interval)										
		Main Estimate	Men	Women	Initial Agreement	Prior Heart Disease	No Prior Heart Disease	Injury Controls	Cancer Controls	Respiratory Controls	SES Adjustment	Clustering Adjustment
Ischemic Heart Disease	Hypertension Alone	0.6 (0.5, 0.6)	0.5 (0.5, 0.6)	0.6 (0.5, 0.7)	0.6 (0.5, 0.6)	0.6 (0.4, 0.8)	0.6 (0.5, 0.7)	1.5 (1.1, 2.3)	0.4 (0.3, 0.5)	0.6 (0.5, 0.7)	0.6 (0.5, 0.6)	0.6 (0.5, 0.7)
	Diabetes Alone	1.1 (0.8, 1.5)	1.2 (0.8, 1.7)	N/A	1.1 (0.8, 1.5)	0.8 (0.3, 2.1)	1.1 (0.8, 1.5)	2.3 (1.3, 4.4)	0.8 (0.5, 1.2)	1.2 (0.8, 1.8)	1.1 (0.8, 1.5)	1.2 (0.8, 1.6)
	Hypertension and Diabetes	0.7 (0.5, 0.8)	0.7 (0.5, 0.9)	N/A	0.6 (0.5, 0.8)	0.7 (0.4, 1.2)	0.7 (0.5, 0.9)	1.3 (0.7, 2.5)	0.4 (0.3, 0.6)	0.8 (0.6, 1.2)	0.7 (0.5, 0.9)	0.7 (0.5, 0.9)
Stroke (high-burden)	Hypertension Alone	0.7 (0.5, 1.0)	0.9 (0.6, 1.4)	0.6 (0.4, 0.9)	0.7 (0.5, 0.9)	0.7 (0.2, 2.2)	0.6 (0.4, 0.8)	2.2 (0.9, 5.4)	0.6 (0.4, 0.9)	0.6 (0.4, 1.0)	0.7 (0.5, 1.0)	0.7 (0.5, 1.0)
	Diabetes Alone	0.8 (0.4, 1.8)	0.8 (0.3, 2.2)	0.6 (0.2, 2.5)	1.1 (0.5, 2.4)	N/A	1.2 (0.4, 3.5)	N/A	0.5 (0.2, 1.3)	N/A	0.9 (0.4, 2.0)	0.9 (0.4, 2.0)
	Hypertension and Diabetes	0.4 (0.2, 0.7)	0.5 (0.2, 1.0)	0.2 (0.1, 0.6)	0.4 (0.2, 0.6)	N/A	0.2 (0.1, 0.5)	N/A	0.2 (0.1, 0.4)	N/A	0.4 (0.2, 0.7)	0.4 (0.2, 0.7)
Stroke (low-burden)	Hypertension Alone	0.9 (0.7, 1.0)	0.8 (0.7, 1.0)	0.9 (0.7, 1.2)	0.9 (0.7, 1.1)	1.1 (0.5, 2.3)	0.7 (0.6, 0.9)	2.2 (1.4, 3.4)	0.6 (0.5, 0.8)	0.9 (0.7, 1.1)	0.8 (0.7, 1.0)	0.8 (0.7, 1.0)
	Diabetes Alone	0.7 (0.5, 1.1)	0.9 (0.5, 1.5)	0.5 (0.3, 1.0)	0.8 (0.5, 1.2)	N/A	0.7 (0.4, 1.1)	1.9 (0.9, 3.9)	0.5 (0.3, 0.9)	0.7 (0.4, 1.2)	0.8 (0.5, 1.2)	0.8 (0.5, 1.2)
	Hypertension and Diabetes	0.8 (0.6, 1.1)	0.7 (0.4, 1.1)	N/A	0.8 (0.5, 1.1)	0.9 (0.3, 2.7)	0.6 (0.4, 1.0)	1.8 (0.8, 3.9)	0.6 (0.4, 0.9)	0.8 (0.5, 1.3)	0.8 (0.6, 1.2)	0.8 (0.6, 1.2)

Abbreviations: N/A, result could not be estimated due to sample size limitations

Appendix References

1. Kumar R, Thakur JS, Rao BT, Singh MMC, Bhatia SPS. Validity of verbal autopsy in determining causes of adult deaths. *Indian J Public Health*. 2006;50(2):90-94.

2. Ke C, Gupta R, Xavier D, et al. Divergent trends in ischaemic heart disease and stroke mortality in India from 2000 to 2015: a nationally representative mortality study. *The Lancet Global Health*. 2018;6(8):e914-e923. doi:10.1016/S2214-109X(18)30242-0
